# Oral microbiome dynamics across periodontitis severity stratified by type 2 diabetes status

**DOI:** 10.1080/20002297.2026.2698205

**Published:** 2026-07-08

**Authors:** Jun Mi, Mengfan Zhi, Xiaoyu Sun, Meihui Li, Tianyong Sun, Xiufeng Gu, Pishan Yang, Qiang Feng

**Affiliations:** a Department of Human Microbiome, School and Hospital of Stomatology, Cheeloo College of Medicine, Shandong University & Shandong Key Laboratory of Oral Tissue Regeneration, Jinan, China; b College & Hospital of Stomatology, Anhui Medical University, Key Lab. of Oral Diseases Research of Anhui Province, Hefei, China; c College of Life Sciences, Northwest University, Xi’an, China; d Department of Periodontology, School and Hospital of Stomatology, Cheeloo College of Medicine, Shandong University & Shandong Key Laboratory of Oral Tissue Regeneration, Jinan, China; e Shandong University-BOP Joint Oral Microbiome Laboratory, Jinan, China; f School of Stomatology, College of Life Sciences and Medicine, Northwest University, Xi’an, China

**Keywords:** Periodontal diseases, systemic health/disease, microbial change, 16S rRNA, oral niche

## Abstract

**Background:**

The oral microbiome serves as an effector of bidirectional promotion between periodontitis and type 2 diabetes mellitus (T2DM). However, the association between diabetes status and oral microbiota alterations and whether these patterns explained increased periodontitis severity remains unclearly illuminated.

**Objective:**

In this study, an investigation was conducted into the association of T2DM with periodontitis severity from the perspective of the oral microbiome.

**Methods:**

This cross-sectional study enrolled cohorts of patients with and without T2DM presenting periodontitis. Combined with bioinformatics and statistical analyses, 16S rRNA gene sequencing was utilized for characterizing the oral microbiome across four oral niches in patients with different T2DM statuses and periodontitis severity.

**Results:**

Oral microbiome composition was dysregulated in the context of periodontitis with or without T2DM. The variation pattern of the oral microbiome showed obvious differences. *Capnocytophaga sputigena*, *Fusobacterium hwasookii,* and *Capnocytophaga gingivalis* demonstrated a significant down-regulation exclusively in T2DM subjects. Compared with non-diabetic (ND) subjects, T2DM subjects exhibited markedly altered correlation patterns between *Filifactor alocis*, *Fusobacterium nucleatum,* and other periodontitis-associated differential microbes and clinical parameters. *Solobacterium moorei*, *Catonella morbi,* and several additional taxa were potential biomarkers of periodontitis severity in T2DM subjects. In addition, T2DM altered microbial interaction between plaque (Pla) and gingival crevicular fluid (GCF) communities, which may form an oral microbial environment facilitating periodontitis severity.

**Conclusion:**

T2DM greatly reshapes periodontitis-associated oral microbial dysbiosis patterns, which additionally display T2DM-specific microbial traits. This highlights the unique regulatory role and significant impact of T2DM on oral microbiome alterations in periodontitis.

## Introduction

Accumulating evidence has corroborated that periodontitis constitutes a prominent risk factor for T2DM onset and progression, while T2DM complications substantially influence periodontitis severity [1,2]. The interconnected relationship between periodontitis and T2DM complications was formally recognized by the European Federation of Periodontology (EFP) and the International Diabetes Federation (IDF) in 2017 [3]. Periodontitis is inherently initiated by a chronic inflammation mediated by oral microbiome dysbiosis [4]. Also, oral microbiome dysbiosis is recognized as the bridging mediator linking periodontitis pathogenesis and T2DM [5,6]. For instance, non-surgical periodontal therapy (NSPT) reduces subgingival bacterial load, which thereby potentially improves glycemic control in T2DM patients [7,8]. Optimal glycemic control enhances the clinical outcomes of NSPT in T2DM patients to a large extent [9,10]. To date, the knowledge of how T2DM contributes to periodontitis severity remains relatively scarce.

Existing studies have reported a series of differentiated oral microbial profiles in periodontitis with/without T2DM at the subgingiva or saliva (Sal) niche. For example, patients with T2DM and periodontitis displayed a drastic reduction in subgingival microbial biodiversity. This is characterized by the decreased levels of anaerobic bacteria and the elevated levels of facultative bacteria [11], along with the increasing abundance of classic periodontal pathogens [12]. Stratified by T2DM status and periodontitis severity, salivary microbiome analysis revealed a series of genera-level variations in periodontitis patients with T2DM and identified potential salivary microbial biomarkers for the early warning of T2DM [13]. Also, T2DM can accelerate periodontitis severity through increasing the abundance of a group of periodontal pathogens while reducing the abundance of other periodontal pathogens [14]. These studies offer key insights into the dysbiosis of the oral microbiome in periodontitis with/without T2DM. Nevertheless, how differentiated oral microbial profiles induced by T2DM increased periodontitis severity is still unclear.

To characterize how T2DM-altered oral bacteria contributed to periodontitis severity, this study profiled the microbial communities of plaque (Pla), gingival crevicular fluid (GCF), tongue back (TB) and saliva (Sal) from healthy individuals (ND-H), patients with moderate or severe periodontitis (ND-MP or ND-SP), T2DM patients without periodontitis (T2DM-H), and T2DM patients with moderate or severe periodontitis patients (T2DM-MP or T2DM-SP). The dynamic shifts of the oral microbiome in multiple oral niches during the progression of periodontitis alone and T2DM-associated periodontitis were investigated. Subsequently, the similarities and divergences among the differentially abundant oral microbiome between these two clinical conditions were compared. By constructing co-expression networks of differentially abundant oral microbiome, the mechanisms by which T2DM-induced microbial perturbations drive periodontitis progression were revealed. Through analyzing differentiated oral microbial profiles before and after interventions targeting periodontitis and T2DM, the impacts of NSPT and glycemic control on the oral microbiome were further elucidated.

## Materials and methods

### Human subjects and ethical approval

This cross-sectional study enrolled cohorts of patients with and without T2DM presenting periodontitis. A total of 185 participants were recruited. Briefly, study information, including the research objectives, significance and potential benefits, was publicized to eligible individuals across local healthcare institutions, comprising two general hospitals, one specialized stomatological hospital, and multiple community health service centers. Individuals who volunteered to participate were pre-scheduled for screening and sampling at designated hospitals by professional researchers. All participants provided written informed consent after fully understanding the study procedures, risks, and benefits. Subsequently, standardized clinical examinations, baseline data collection, and biological specimen acquisition were performed uniformly by a professional research team. The inclusion criteria were as follows: (1) non-T2DM healthy individuals (ND-H: healthy subjects without periodontitis, T2DM, or other systemic diseases who underwent health check-ups within one year prior to this study), patients with moderate or severe periodontitis without T2DM (ND-MP or ND-SP), T2DM patients without periodontitis (T2DM-H), T2DM patients with moderate or severe periodontitis (T2DM-MP or T2DM-SP), and T2DM patients with controlled glycaemia; (2) patients aged from 18 to 80 upon enrollment; and (3) patients providing signed informed consent. The exclusion criteria were as follows: (1) patients treated with antibiotics within three months or those who received periodontal treatment within six months; (2) pregnant or lactating women; (3) current smokers; and (4) patients with other systemic diseases (e.g. pulmonary, cardiovascular, liver and/or cerebral diseases). The current study gained the approval of the Ethics Committee of Anhui Medical University for Nationalities (No. 20180163). It was performed as per the Declaration of Helsinki. This trial was registered in the Chinese Clinical Trial Registry (Registration number: ChiCTR1800015652). Periodontitis was diagnosed according to the criteria of the 1999 International Classification for periodontal diseases [15]. Of note, all clinical samples and examinations were conducted in 2018. The new 2017 classification system had just been issued, but was not widely used in clinical practice that year. Therefore, the 1999 classification was adopted to ensure diagnostic consistency and reproducibility during sample collection. Briefly, patients with moderate periodontitis had a probing depth (PD) of ≤6 mm and a clinical attachment level (CAL) of 3-4 mm, and severe periodontitis patients had a PD of >6 mm and a CAL of ≥5 mm. T2DM was confirmed by a fasting blood glucose of >7.0 mmol/L and a hemoglobin A1c (HbA1c) concentration of >7%, as specified by the American Diabetes Association in 2018 [16]. T2DM patients with controlled glycemia enrolled in this study included individuals with a definitive history of type 2 diabetes mellitus (T2DM) with well-controlled glycemic status, defined as fasting blood glucose <7.0 mmol/L and glycosylated hemoglobin (HbA1c) <7%. Notably, not all diabetic patients were newly diagnosed. All enrolled patients had confirmed T2DM diagnosis prior to sample collection, and their glycemic control indicators were strictly evaluated and recorded at the enrollment time point. All oral samples were gleaned at baseline before any periodontal treatment. None of the subjects had received periodontal therapy, including scaling, root planing, and surgical treatment, within six months before sample collection. The purpose was to avoid the influence of clinical intervention on the oral microbiome profile. This study was designed as a cross-sectional observational study, and no clinical follow-up or long-term outcome tracking was conducted for all enrolled participants.

### Sample collection and preparation

All participants were enrolled after providing a written informed consent form. They were instructed to refrain from eating, drinking, and performing oral hygiene procedures for two hours before sampling. The oral and dental health statuses of each participant were assessed by calibrated dentists. Subgingival Pla and GCF were collected from the site with the maximum full-mouth PD at baseline. Both samples were obtained from the identical site of the same tooth for each participant.

Before sampling, participants rinsed their oral cavity with sterile water for 1 min. A sterile supragingival scaler was used to remove supragingival Pla. Then, the tooth surface was rinsed with normal saline and air-dried. Next, cotton rolls were placed for moisture isolation.

First, GCF sampling was performed using 3 M Waterman No. 1 filter strips (a width of 2 mm, a length of 1 cm). The filter strips were gently inserted into periodontal pockets, retained for 1 min and subsequently transferred into Eppendorf (EP) tubes containing 0.5  mL of 1 × phosphate buffered saline (PBS). All GCF samples were stored at −80 °C until further analysis.

Subsequently, sterile Gracey curettes were leveraged to harvest subgingival Pla. They were carefully inserted into periodontal pockets. Subgingival Pla was scraped from the pocket base toward the crown. The collected Pla was immediately placed into EP tubes preloaded with 0.5  mL of 1 × PBS. The samples were centrifuged at 3,000  rpm for 5 min. The supernatant was discarded. The pellets were stored at −80 °C. For the healthy control group, GCF and Pla samples were gathered from sites with PD <2 mm, no bleeding on probing, and no gingival erythema or swelling. All sampling procedures were performed gently and expeditiously with minimal repeated probing in order to prevent the blood contamination of samples.

Unstimulated whole Sal was collected via the passive drool method. Participants rinsed their mouths with sterile water for 1 min and then tilted their heads slightly downward with the mouth slightly open. In total, 0.5–1 mL of unstimulated Sal was collected into EP tubes. The samples were subjected to 10- to 20-s vortexing to disperse bacterial aggregates and stored at −80 °C.

Dorsal tongue Pla was collected using sterile swabs by gently scraping the dorsal tongue surface 10 times. The swabs with Pla samples were placed into EP tubes with 1  mL of 1 × PBS and preserved at −80 °C.

### Deoxyribonucleic acid extraction and sequencing of Pla, GCF, Sal and TB

For high DNA purity, the cetyltrimethylammonium bromide (CTAB) method has been used, which briefly involves a proteinase K treatment, CTAB, and NaCl to precipitate proteins, followed by a chloroform-isoamyl alcohol extraction [17]. After extracting the genomic DNA of all samples, the V3–V4 hypervariable regions of the 16S rRNA gene were amplified by polymerase chain reaction (PCR) and then sequenced on HiSeq 2500 platform. Raw sequencing reads were processed using the DADA2 pipeline for quality control, denoising, and inference of amplicon sequence variants (ASVs), followed by the removal of chimeric sequences. Taxonomic assignment was conducted based on the SILVA database (version 138.2). The obtained ASVs were further filtered by removing those detected in fewer than 20% of the samples. After filtering, a total of 3,098 ASVs were retained and included in subsequent analyses.

### Statistical analysis

RStudio (version 4.3.0) was applied to conduct statistical analyses and visualize the data. Stacked bar and pie charts were utilized for the visualization of the top 10 most abundant bacterial phyla. To assess differences in community structure in light of the Bray-Curtis distance, principal coordinates analysis (PCoA) was performed using the ‘pcoa’ function from the ‘ape’ package (version 5.8-1). Based on the Bray–Curtis distance, the Adonis2 method implemented in the ‘vegan’ package (version 2.6-6.1) was adopted to evaluate the statistical significance of community differences. R^2^ and *p*-values were reported to quantify the proportion of variance explained by grouping factors and their significance. Spearman correlation analysis was performed to analyze the correlations between alpha diversity and clinical indicators. The Spearman correlation analysis is implemented using the ‘cor.test’ function from the ‘stats’ package.

Microbial community comparisons among populations were performed by calculating pseudo-F values using the R package ‘vegan’ (version 2.6-6.1). The Wilcoxon test was used to evaluate whether group differences shown in boxplots were significant. The R package MaAsLin2 (version 2_1.14.1) was utilized to conduct differential analysis between different cohorts. Potential confounding factors, including age, gender and body mass index (BMI), were adjusted in the model. Microbes with *p*-values < 0.05 were considered to show significant differences. Subsequently, Spearman's partial correlation analysis was applied to evaluate the relationships of differential microbes with clinical indicators. Age, gender and BMI were also adjusted in the model. Partial correlation analyses were performed using the ‘pcor.test’ function from the R package ‘ppcor’ (version 1.1). Furthermore, mediation analysis was carried out to explore the potential mediating effects among differential microbes, clinical indicators and periodontitis progression utilizing the ‘mediate’ function from the R package ‘mediation’ (version 4.5.0) [18]. Age, gender and BMI were also adjusted. A significant mediation effect was defined to have both ACME *p*-value < 0.05 and total effect *p*-value < 0.05. Spearman correlation analysis was used to calculate the correlations between differential microbes. Microbial interaction networks for each population were constructed based on thresholds (|correlation coefficient| ≥ 0.4, *p* < 0.05). Network structures were visualized using Gephi (version 0.9.2) and Cytoscape software (version 3.7.1). The network dissimilarity index was calculated to quantify the differences between networks of different populations [19,20]. Additionally, whether the network of each population was robust was evaluated separately [21]. Robustness was computed by simulating random node removal: it was recalculated after removing 5% of nodes at each iteration. The Wilcoxon test was used to assess significant differences in the robustness of networks among different populations. For the purpose of identifying potential key ‘driver’ nodes, the NetMoss score of each network node was calculated using the ‘NetMoss’ function from the R package NetMoss2 (version 0.2.0) [22]. Nodes that had *p* < 0.05 and ranked among the top 10 in NetMoss scores were regarded as key nodes. The NetMoss score reflects the ‘network-driven factors’ based on the importance of the network topology structure. The Fast Expectation-Maximization for Microbial Source Tracking (FEAST) method was employed to perform the source tracking analysis of microbial compositions across four oral ecological niches in diverse population groups [23]. The FEAST analysis was performed with the R package ‘FEAST’ (version 1.10.0). Moreover, the phylogenetic bin-based null model analysis (iCAMP) was utilized for evaluating the relative contributions of stochastic and deterministic processes in the assembly of oral microbial communities [24]. The qpen function in the R package ‘iCAMP’ (version 1.5.12) was used to conduct this analysis.

## Results

### Characteristics of the oral microbiome in healthy and periodontitis subjects with or without T2DM

**Figure 1.**
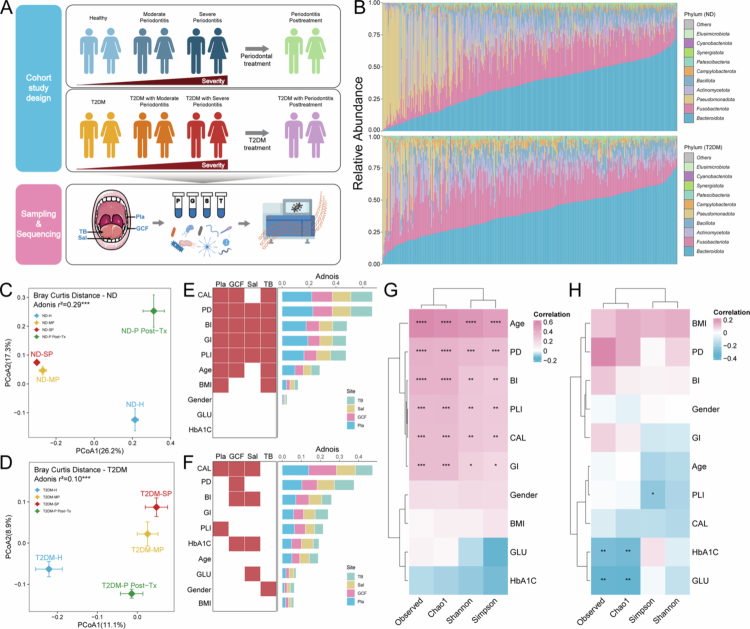
Information on community structure and microbial composition of the oral microbiome in healthy and periodontitis groups with and without T2DM. (A) A total of 772 samples were collected from four oral niches (supragingival plaque, gingival crevicular fluid, saliva, and tongue dorsum) across eight population groups. (B) Stacked bar plots showing the relative abundances of the top 10 bacterial phyla in all individual samples from ND and T2DM subjects. All samples are arranged in ascending order according to the relative abundance of the phylum Bacteroidota. ‘Other’ represents the remaining taxa. (C) PCoA based on Bray–Curtis distance showing community differences among the ND-H, ND-MP, ND-SP, and ND*-*P Post-tx groups at the Pla niche. (D) PCoA based on Bray-Curtis distance showing community differences among the T2DM-H, T2DM-MP, T2DM-SP and T2DM-P Post-tx groups at the Pla niche. (E) Adonis analysis of the associations between clinical parameters and microbial communities across four oral ecological niches in ND subjects. In the heatmap on the left, red indicates Adonis *p*-values < 0.05. (F) Adonis analysis of the associations between clinical parameters and microbial communities across four oral ecological niches in T2DM subjects. In the heatmap on the left, red indicates Adonis *p*-values < 0.05. (G) The correlations between clinical parameters and alpha diversity indices of the Pla niche in ND subjects. (H) The correlations between clinical parameters and alpha diversity indices of the Pla niche in T2DM subjects. *p-value < 0.05, **p-value < 0.01, ***p-value < 0.001, ****p-value < 0.0001.

To characterize oral microbiome alterations during periodontitis progression in non-diabetic (ND) or T2DM subjects, 772 oral microbiome samples were collected and analyzed. They included 196 subgingival Pla, 183 GCF, 196 Sal and 197 TB samples obtained from 50 periodontally healthy subjects (ND-H), 19 subjects with moderate periodontitis (ND-MP, including four revisits), 31 subjects with severe periodontitis (ND-SP, including eight revisits), nine subjects with T2DM but without periodontitis (T2DM-H), 22 subjects with T2DM and periodontitis receiving glycemic-control treatment, 23 subjects with T2DM and moderate periodontitis (T2DM-MP), and 31 subjects with T2DM and severe periodontitis (T2DM-SP) (Figure 1A, Appendix Table 1). All clean reads were processed using the DADA2 pipeline. A total of 3,098 ASVs were generated and assigned to 17 phyla, 25 classes, 60 orders, 86 families, 185 species and 200 genera.

An analysis was made of major microbial compositions at the phylum level in ND and T2DM subjects with varying periodontitis statuses. As shown in Figure 1B, the top three dominant phyla were *Bacteroidota*, *Fusobacteriota* and *Pseudomonadota* in ND subjects and *Bacteroidota*, *Fusobacteriota* and *Actinomycetota* in T2DM subjects. The composition and ranking of dominant phyla varied substantially across oral niches and periodontitis stages (Appendix Figures 1A–D). Beyond that, *β*-diversity across different periodontitis statuses in ND and T2DM subjects was evaluated. PCoA based on the Bray-Curtis distance revealed significant separation in community structures among samples with different periodontitis statuses at the Pla niche in ND and T2DM subjects (ND: Adonis *r* ^2^ = 0.29***; T2DM: Adonis *r* ^2^ = 0.10***) (Figures 1C–D). Similar trends were also observed in other oral niches (Appendix Figures 2A–F). Next, Adonis analyses were performed to evaluate the associations between host clinical parameters and the four oral niches. In ND subjects, the Pla microbiota was most strongly correlated with most clinical parameters. Microbial communities in all niches except Sal displayed a significant association with periodontal indices (Figure 1E). In T2DM subjects, the GCF microbiota was most significantly associated with periodontal indices, followed by Pla and Sal microbiota, while the TB microbiota was only significantly related to gender (Figure 1F). Moreover, HbA1c, the T2DM-related index, was closely correlated with both GCF and Sal microbes, whereas glutamic acid (GLU) was primarily associated with the Sal microbiome (Figure 1F). Correlation analysis between clinical parameters and *α*-diversity revealed that in ND subjects, *α*-diversity indices at the Pla niche were significantly and positively correlated with age and periodontal indices (Figure 1G), a pattern also observed in the Sal niche. In GCF and TB niches, *α*-diversity indices exhibited a negative correlation with age and periodontal indices (Appendix Figures 2G–I). In T2DM subjects, Observed and Chao1 indices at the Pla niche demonstrated a significant negative correlation with HbA1c and GLU (Figure 1H). In the GCF and Sal niches, however, *α*-diversity indices were positively correlated with HbA1c and GLU (Appendix Figures 2J–L). Collectively, these findings indicate that overall microbial characteristics differed substantially between ND and T2DM subjects across varying periodontitis conditions.

**Figure 2.**
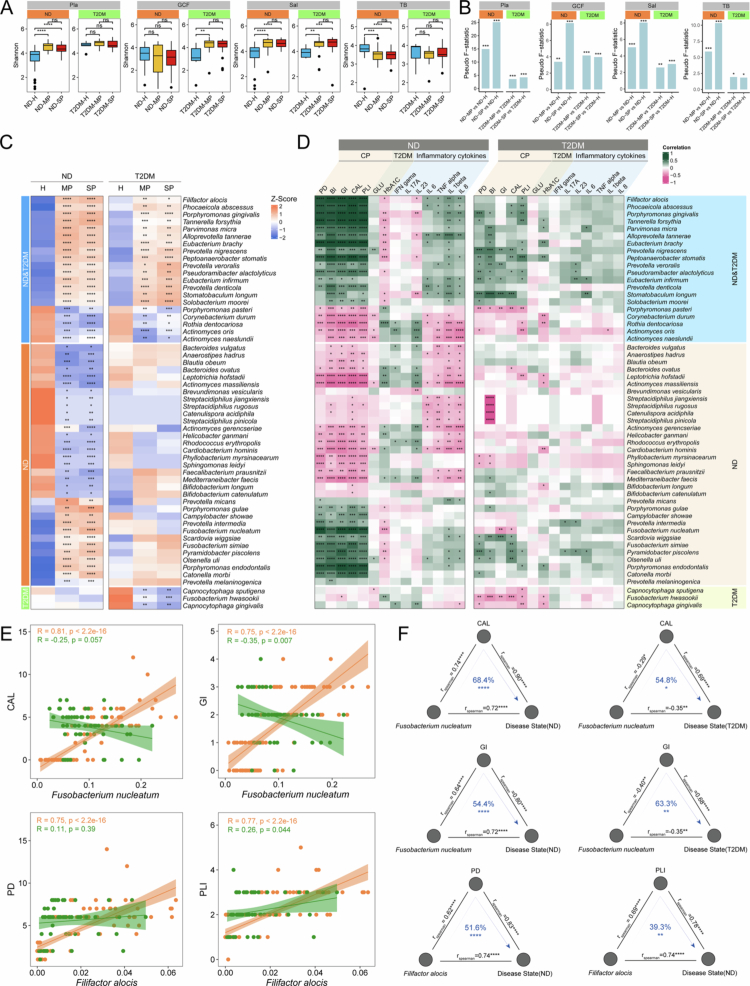
Alterations in the oral microbial composition and its correlations with clinical parameters in healthy and periodontitis groups with and without T2DM. (A) Boxplots showing the distribution and intergroup comparisons of Shannon diversity among healthy, moderate periodontitis, and severe periodontitis groups in ND and T2DM subjects. (B) Bar plots showing the pseudo-F values of microbial communities between the healthy group and the moderate or severe periodontitis groups in ND and T2DM subjects. (C) Heatmaps of microbial taxa showing significant differences between moderate or severe periodontitis and healthy groups in the Pla niche of ND and T2DM subjects. Microbes were classified according to the following criteria: taxa showing significant differences only in ND subjects, only in T2DM subjects, and in both ND and T2DM subjects. (D) Results of correlation analysis between clinical parameters of ND and T2DM subjects and differential species in the Pla niche (Figure c), respectively. (E) Regression analysis of the associations between Fusobacterium nucleatum/Filifactor alocis and selected clinical parameters at the Pla niche in ND and T2DM subjects. (F) Mediation analysis of the associations among Fusobacterium nucleatum/Filifactor alocis, clinical parameters, and periodontitis progression at the Pla niche in ND and T2DM subjects. *p-value < 0.05, **p-value < 0.01, ***p-value < 0.001, ****p-value < 0.0001.

### Oral microbe alterations in periodontitis patients with or without T2DM

Comparisons were made between *α*-diversity indices across oral niches under different periodontal conditions in order to characterize the differentiated oral microbial profiles with the higher periodontal disease severity in subjects with or without T2DM. In ND subjects, individuals with periodontitis had significantly higher four *α*-diversity indices at Pla and Sal niches than healthy controls (Figure 2A; Appendix Figures 3A and C), whereas Observed and Chao1 indices at the GCF niche were significantly reduced (Appendix Figure 3B). At the TB niche, Shannon and Simpson indices also presented a significant decreasing trend (Figure 2A; Appendix Figure 3D). In T2DM subjects, periodontitis patients demonstrated a marked increase in all four *α*-diversity indices at GCF and Sal niches compared with healthy individuals (Figure 2A; Appendix Figures 3B–C). The pseudo F-statistic results revealed that non-periodontitis and periodontitis individuals showed significant differences in microbial community structures in ND and T2DM conditions (Figure 2B). Moreover, the Pla niche exhibited remarkably higher Pseudo F-statistic values than the other oral niches. This suggests the central role of the Pla microbial ecosystem in periodontitis conditions.

**Figure 3.**
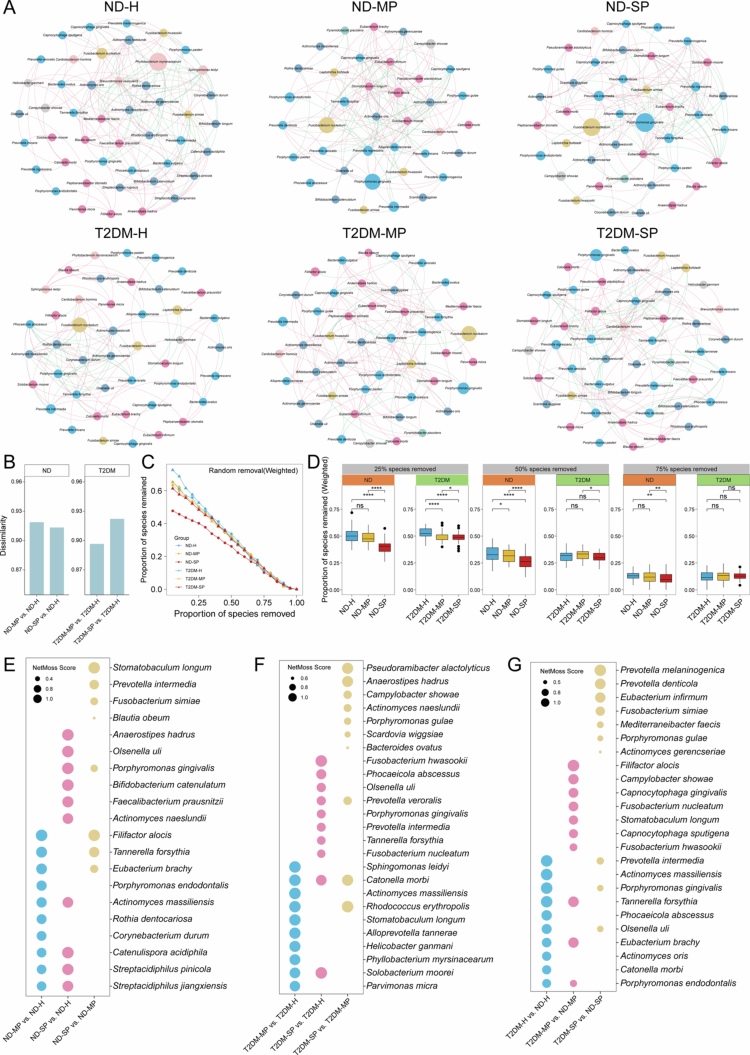
Co-occurrence network and network property analysis of differential microbes in Pla niche. (A) Co-occurrence network analysis of differential microbes in Pla niche. (B) Network dissimilarity index between the healthy group and the moderate or severe periodontitis networks in ND and T2DM subjects. (C) Line plots showing the robustness values of each microbial co-occurrence network. (D) Comparison of network robustness among groups after removing 25%, 50% and 75% of nodes from the co-occurrence networks. (E) Top 10 microbial taxa ranked by NetMoss scores in ND subjects. (F) Top 10 microbial taxa ranked by NetMoss scores in T2DM subjects. (G) Top 10 microbial taxa ranked by NetMoss scores obtained from NetMoss analysis between ND and T2DM subjects under the same periodontitis status. *p-value < 0.05, **p-value < 0.01, ****p-value < 0.0001.

To examine oral microbial alterations associated with the conditions of periodontitis in ND and T2DM subjects, differential abundance analyses were performed between healthy individuals and patients with moderate or severe periodontitis using the MaAsLin2 method. Confounding factors such as age, gender and BMI were adjusted. Microbes showing significant differences in both moderate and severe periodontitis groups compared to healthy controls were defined as periodontitis-related differential taxa. Under the ND condition, 17, 29, 39 and 53 differential species were identified in TB, Sal, GCF and Pla niches, respectively. Under the T2DM condition, 23, 29, 20 and 1 differential species were identified across these niches (Figure 2C; Appendix Figs. S4A-C; Appendix Table 2). At the Pla niche, 20 species were identified as periodontitis-related differential microbes shared between both ND and T2DM subjects. These encompassed *Tannerella forsythia*, *Porphyromonas gingivalis* and *Parvimonas micra* from the ‘red’ or ‘orange’ complex, as well as established periodontal pathogens *F. alocis* and *Eubacterium brachy* [25,26] (Figure 2C). Among 33 ND-specific differential species, typical periodontal pathogens, including *F. nucleatum*, *Prevotella intermedia*, *Porphyromonas endodontalis* and *Olsenella uli,* displayed marked enrichment in patients with periodontitis [27,28] (Figure 2C). In contrast, *Bifidobacterium longum* and *Bifidobacterium catenulatum*, which contribute to gut microbial homeostasis, exhibited a notable reduction in the periodontitis group (Figure 2C) [29,30]. In T2DM subjects, only three T2DM-specific differential species: *C. sputigena*, *F. hwasookii* and *C. gingivalis*, were identified, which all demonstrated reduced abundance in the periodontitis group. In GCF and Sal niches, *T. forsythia*, *P. micra*, *F. alocis* and *E. brachy* were consistently identified as differential species associated with periodontitis in ND and T2DM subjects (Appendix Figure 4A–B). Fewer periodontitis-associated species were detected at the TB niche, although a few key periodontal pathogens such as *T. forsythia*, *P. gingivalis* and *F. nucleatum* remained identifiable (Appendix Figure 4C).

**Figure 4.**
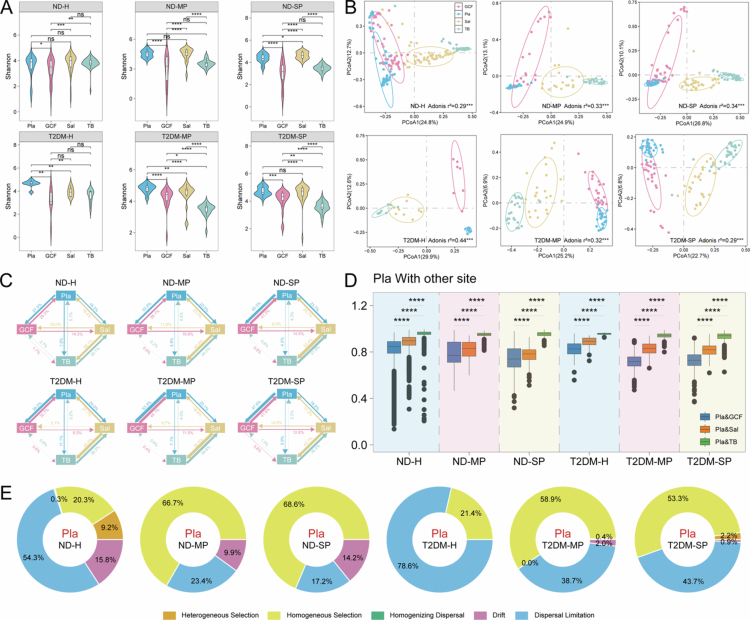
Analysis of microbial source tracking and community assembly mechanisms across oral ecological niches under different periodontitis and T2DM conditions. (A) Violin plots showing the distribution of Shannon diversity across oral ecological niches under different periodontitis and T2DM conditions. (B) PCoA based on Bray–Curtis distances showing the microbial community differences among four oral ecological niches under different periodontal and T2DM conditions. (C) FEAST source tracking analysis among the four oral ecological niches under different periodontitis and T2DM statuses. The thickness of the arrows and the annotated values indicate the proportion of source tracking between the two ecological niches. (D) Bray–Curtis distances between the Pla niche and other oral ecological niches across different periodontitis and T2DM statuses. (E) Contributions of deterministic and stochastic processes to community assembly mechanisms in the Pla niche under different periodontitis and T2DM statuses. *p-value < 0.05, **p-value < 0.01, ***p-value < 0.001, ****p-value < 0.0001.

We performed correlation analyses between the differential species and clinical parameters, as well as with inflammatory factors. Subsequently, at the Pla niche, species up-regulated in periodontitis were predominantly positively correlated with periodontal indices, whereas species down-regulated in periodontitis were primarily negatively correlated (Figure 2D). Overall, a greater number of significant correlations were noted under the ND condition than under the T2DM condition. It is noteworthy that *F. alocis* showed significant positive correlations with all periodontal indices in ND subjects but a significant association only with PLI in T2DM subjects (Figure 2D). *F. nucleatum* exhibited positive correlations with periodontal indices under the ND condition but shifting to negative correlations under the T2DM condition, as further supported by regression analyses (Figure 2E). Similar trends were noticed in GCF, Sal and TB niches. In these niches, more significant associations were detected under the ND condition than under the T2DM condition (Appendix Figures 5A–C).

**Figure 5.**
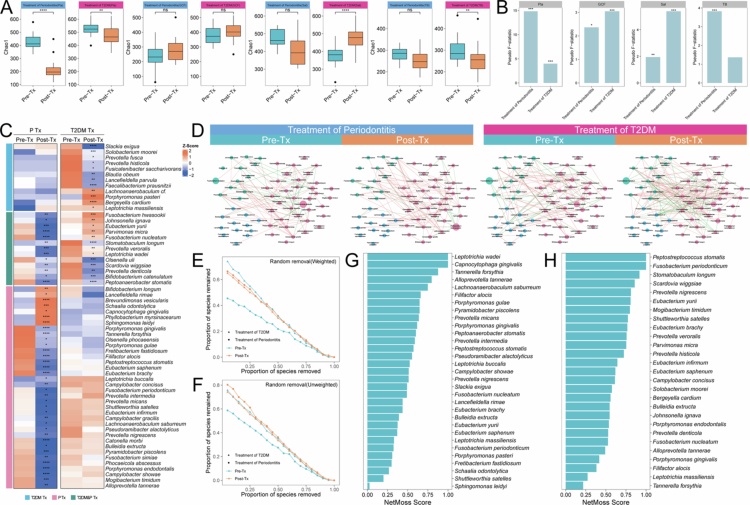
Impacts of periodontal and T2DM treatments on differentiated oral microbial profiles and network characteristics. (A) Boxplots illustrating the distribution of the Chao1 diversity index before and after periodontal treatment, as well as before and after T2DM treatment. (B) Bar plots showing the pseudo-F values of microbial communities before and after periodontal treatment and before and after T2DM treatment. (C) Heatmaps of microbial taxa significant differences between before and after treatment in the Pla niche of periodontal treatment and T2DM treatment. Microbes were classified according to the following criteria: taxa showing significant differences only in periodontal treatment, only in T2DM treatment, and in both periodontal treatment and T2DM treatment. (D) Co-occurrence network analysis of differential microbes in Pla niche. (E) Line plots showing the robustness(Weighted) values of each microbial co-occurrence network. (F) Line plots showing the robustness(Unweighted) values of each microbial co-occurrence network. (G) NetMoss scores of co-occurrence networks before and after periodontal treatment, with significant scores (*p* < 0.05) ranked in descending order. (H) NetMoss scores of co-occurrence networks before and after T2DM treatment, with significant scores (*p* < 0.05) ranked in descending order. *p-value < 0.05, **p-value < 0.01, ***p-value < 0.001, ****p-value < 0.0001.

Then, mediation analyses were conducted to evaluate potential mediating relationships among microbes, clinical parameters and periodontitis severity. In total, 174 and 40 significant mediation effects were identified under both ND and T2DM conditions, respectively (Appendix Figure 5D–E; Appendix Table 3). In ND subjects, *F. nucleatum* was significantly enriched in periodontitis patients and positively associated with disease severity, but it showed a negative association under the T2DM condition (Figure 2F). *F. alocis* was significantly and positively linked to periodontitis severity in ND subjects, and mediated disease progression through clinical parameters like PD and Pla index (PLI). However, no significant mediation relationships were found in T2DM subjects (Figure 2F; Appendix Figure 5D–F). Collectively, these findings uncover significant mediating relationships between differential microbes and clinical parameters, which suggests that these microbes may play a key role in periodontitis progression.

### Differential alterations in Pla microbial co-occurrence networks during periodontitis progression with or without T2DM

Differential microbial co-occurrence networks for each population were constructed to explore how periodontitis severity affects Pla microbial interactions under ND and T2DM conditions (Figure 3A). Network dissimilarity values exceeded 0.84 under the above two conditions. Values greater than 0.91 were observed in ND subjects (Figure 3B). Next, network robustness under random node removal was assessed by simulating species extinction. Under the ND condition, network robustness displayed a decreasing trend with the increase of periodontitis severity (Figure 3C, D; Appendix Figure 6A, B). Under the T2DM condition, when 25% of nodes were removed, subjects with periodontitis and T2DM had significantly lower network robustness than T2DM-only subjects (Figure 3C, D). These results indicate that periodontitis severity and T2DM condition markedly reshape Pla microbial interaction networks and compromise their structural stability.

To identify potential biomarkers of the transition from health to periodontitis under ND and T2DM conditions, the NetMoss method was adopted to compare microbial co-occurrence networks between non-periodontitis subjects and subjects with moderate or severe periodontitis. The NetMoss score was calculated for each node, and significant potential candidate taxa were chosen based on *p* < 0.05 and the top 10 NetMoss scores. The top three potential biomarkers between the ND-H and ND-MP groups were periodontal pathogens including *T. forsythia*, *F. alocis* and *E. brachy* (Figure 3E). For the comparison of ND-H and ND-SP, the top biomarkers contained the red-complex member *P. gingivalis*, along with *O. uli* and *Anaerostipes hadrus* (Figure 3E). Shared potential biomarkers between ND-H vs. ND-MP and ND-H vs. ND-SP comparisons included *Actinomyces massiliensis*, *Catenulispora acidiphila*, *Streptacidiphilus pinicola* and *Streptacidiphilus jiangxiensis*. All of them showed a remarkable down-regulation in periodontitis (Figure 3E). Notably, typical periodontal pathogens *T. forsythia*, *P. gingivalis* and *F. alocis* were also confirmed as potential biomarkers for the comparison of ND-MP vs. ND-SP. Under the T2DM condition, the top three potential candidate taxa for the comparison of T2DM-H vs. T2DM-MP were *Sphingomonas leidyi*, *C. morbi* and *A. massiliensis* (Figure 3F). For the comparison of T2DM-H vs. T2DM-SP, *S. moorei*, *F. hwasookii* and *C. morbi* were identified as the top biomarkers. *C. morbi* emerged as a shared potential biomarker across multiple T2DM network comparisons (Figure 3F). Potential biomarkers were further compared between ND and T2DM subjects within the same periodontitis stage. Shared biomarkers between the comparisons of ND-H vs. T2DM-H and ND-MP vs. T2DM-MP included *T. forsythia*, *E. brachy* and *P. endodontalis* (Figure 3G). In severe periodontitis, potential biomarkers between ND-SP and T2DM-SP again included classical periodontal pathogens like *P. gingivalis* and *O uli* (Figure 3G). On balance, these results indicate that T2DM status and periodontitis stage exert substantial association on the interaction of Pla-associated microbial communities.

#### Microbial source contribution and community assembly across oral niches under different periodontitis and T2DM conditions

The *α*- and *β*-diversity of the four oral niches were compared to elucidate alterations in microbial community assembly across oral niches under various periodontitis and T2DM conditions. The results showed that *α*-diversity patterns varied substantially among multiple disease conditions (Figure 4A; Supplementary Figure S7A–C). In subjects with periodontitis with or without T2DM, all four diversity indices at Pla and Sal niches were substantially higher than those at GCF and TB niches. In the ND-H group, Pla and GCF niches exhibited significant differences in the Shannon index (Figure 4A; Appendix Figure 7A–C). Meanwhile, the PCoA results revealed significant separation among oral-niche samples across a variety of disease conditions (Adonis *r* ^2^: ND-H = 0.29, ND-MP = 0.33, ND-SP = 0.34, T2DM-H = 0.44, T2DM-MP = 0.32, T2DM-SP = 0.29) (Figure 4B).

Then, the FEAST method was applied to investigate microbial source contributions across four oral niches in periodontitis patients with or without T2DM. The results showed that the contribution between Pla and GCF progressively increased with the severity of periodontitis under the ND condition. Specifically, the contribution of GCF to the Pla community increased from 25.3% in the ND-H group to 26.1% in the ND-MP group and further to 41.5% in the ND-SP group (Figure 4C). A similar trend was detected between Sal and TB. The contribution of TB to the Sal community rose from 30.7% in the ND-H group to 34.2% in the ND-MP group, and reached 41.8% in the ND-SP group (Figure 4C). Under the T2DM condition, the source contribution from GCF to Pla was also substantially higher in T2DM-MP and T2DM-SP groups than in the T2DM-H group (Figure 4C). Moreover, the contribution of GCF to Pla was consistently higher in T2DM subjects than in ND subjects within the same periodontitis stage (Figure 4C). Next, similarities in microbial community composition among oral niches were examined based on the Bray-Curtis distance. Under ND and T2DM conditions, and regardless of periodontitis status, the community distance between Pla and GCF was well below their distances to other niches (Figure 4D; Appendix Figure 8A). Likewise, the community distance between Sal and TB was far below their distances to the remaining niches (Appendix Figures 8B, C). These findings indicate that microbial exchange and community similarity among distinct oral niches increase during increased severity of periodontitis. It is a trend further exacerbated in the presence of T2DM.

The phylogenetic bin-based null model analysis (iCAMP) was utilized for exploring the assembly mechanisms of microbial communities across oral niches and quantifying the contributions of stochastic and deterministic processes to oral community assembly. Deterministic processes included heterogeneous and homogeneous selection (HeS and HoS), whereas stochastic ones comprised dispersal limitation (DL), drift (DR) and homogenizing dispersal (HD). In ND subjects, community assembly at the Pla niche in the ND-H group was mainly driven by stochastic processes (DL: 54.3%, HD: 0.3%, DR: 15.8%). The enhanced deterministic assembly process suggests that microbial communities under disease conditions may be shaped by stronger environmental filtering or niche selection. By comparison, assembly shifted toward deterministic processes in ND-MP and ND-SP groups, with HoS increasing to 66.7% and 68.6%, respectively (Figure 4E). A similar pattern was detected under the T2DM condition, where higher periodontal disease severity was associated with reduced DL and increased HoS. In contrast, periodontitis promoted a shift from deterministic to stochastic community assembly at the TB niche (Appendix Figure 8F). Except for the T2DM-SP group, community assembly at the GCF and Sal niches in all other groups was consistently dominated by stochastic processes at GCF and Sal niches (Appendix Figs. 8D-E). These findings indicated that microbial community similarity and source contributions among oral niches increase substantially with the increase in periodontitis severity and T2DM, while environmental filtering or niche selection within oral sites may also be changed.

### Association of periodontal therapy or T2DM management on microbial communities in different oral niches

The *α*-diversity of microbial communities in the four niches before and after periodontal therapy or T2DM intervention was first compared, respectively, with a view to investigating the association of periodontal and T2DM treatment on microbial communities across distinct oral niches. All diversity indices in the Pla niche decreased after both interventions. A more pronounced reduction was observed after periodontal treatment than after T2DM treatment (Figure 5A; Appendix Figure 9A). Conversely, no significant differences were found in the GCF niche after either treatment (Figure 5A; Appendix Figure 9B). In the Sal niche, all four *α*-diversity indices displayed a marked increase after T2DM treatment (Figure 5A; Appendix Figure 9C). In the TB niche, only Chao1 and Observed indices decreased significantly following T2DM treatment (Figure 5A; Appendix Figure 9D). Then, Pseudo F-statistic values were calculated to assess the differences in microbial community structures before and after the two interventions. The results suggested that both periodontal and T2DM treatments induced significant shifts in microbial community structures at Pla, GCF and Sal niches, whereas the TB niche exhibited significant structural differences only after T2DM treatment (Figure 5B).

Subsequently, differential abundance analyses of microbial communities before and after both treatments were conducted using MaAsLin2, and confounders including age, gender and BMI were adjusted. After periodontal treatment, 49, 41, 34 and 14 differentially abundant taxa were identified at Pla, GCF, Sal and TB niches, respectively. However, T2DM treatment resulted in 25, 29, 33 and 7 differentially abundant taxa at corresponding niches (Figure 5C; Appendix Figures 10A–C; Table S4). At the Pla niche, 13 microbes were consistently altered by both interventions, including key periodontal pathogens *F. nucleatum* and *P. micra* (Figure 5C). It should be noted that the abundances of these pathogens decreased significantly after periodontal treatment but increased markedly after T2DM treatment (Figure 5C). In addition, 36 microbes exhibited significant differences exclusively after periodontal treatment. Among them, major periodontal pathogens *P. gingivalis*, *T. forsythia*, *E. brachy* and *Fretibacterium fastidiosum* [31] showed marked reductions in abundance (Figure 5C). Only after T2DM treatment, 12 microbes were significantly altered, including *S. exigua* and *S. moorei* [31,32]. *S. exigua* and *S. moorei* both exhibited significantly decreased abundances after T2DM treatment (Figure 5C). At the GCF niche, *P. gingivalis*, *T. forsythia*, *F. fastidiosum* and *F. alocis* were consistently identified as differentially abundant before and after both treatments. They showed salient reductions in abundance after each intervention (Appendix Figure 10A). At the Sal niche, these same pathogens exhibited significantly reduced abundances only after periodontal treatment (Appendix Figure 10B).

Subsequently, differential microbial co-occurrence networks were constructed at the Pla niche before and after the two treatments to examine the effects of periodontal therapy and T2DM management on oral microbial co-occurrence patterns (Figure 5D). The network dissimilarity index exceeded 0.9 after both treatments. Greater dissimilarity was observed between pre- and post-periodontal treatments than between pre- and post-T2DM treatments (Appendix Figure 10D). Next, network stability was assessed by evaluating the robustness of microbial co-occurrence networks. Periodontal treatment markedly enhanced network robustness. After T2DM treatment, network robustness showed a decrease when 25 or 50% of nodes were removed but a significant increase when 75% of nodes were removed (Figure 5E, F; Appendix Figures 10E, F). The NetMoss method was further applied to compare microbial co-occurrence networks before and after each treatment, and NetMoss scores for all nodes were calculated. Significant nodes (*p* < 0.05) were retained for driver analysis. For periodontal treatment, the top three potential biomarkers were *Leptotrichia wadei*, *C. gingivalis* and *T. forsythia* (Figure 5G). Additional periodontal pathogens, including *P. gingivalis*, *F. nucleatum*, *F. alocis* and *S. exigua* were also identified (Figure 5G). After T2DM treatment, the top three candidate taxa were *Peptostreptococcus stomatis*, *Fusobacterium periodonticum* and *Stomatobaculum longum*. Other periodontitis-associated species like *P. micra* and *E. brachy* were also identified as potential biomarkers (Figure 5H). These findings suggest that periodontal and T2DM treatments exert a significant impact on the structure of oral microbial communities. This underscores the important role of therapeutic interventions in maintaining oral microecological homeostasis.

## Discussion

The previous study of researchers showed the dynamic succession of microbial communities during periodontitis progression, in which the GCF microbial community plays a critical role [33]. Mounting evidence has established that T2DM promotes periodontitis development by altering oral microbiome composition [34]. To date, the different of microbial communities across various oral ecological niches with periodontitis severity under the T2DM condition have not been fully illuminated. This study systematically characterized the patterns of oral microbiome diversity alterations across graded periodontitis severities in individuals with and without T2DM. It was found that correlations between microbial features and periodontal clinical parameters were significantly stronger in ND subjects than in those with T2DM. This observation implies that T2DM reshapes the interaction between the oral microbiome and the local periodontal environment in a systematic way. This is probably ascribed to the T2DM-associated chronic systemic inflammatory state, the accumulation of advanced glycation end products (AGEs), or an altered metabolic environment. These systemic and metabolic disturbances may disrupt the normal microbe-host crosstalk, which leads to weakened correlations under diabetic conditions.

Alterations in oral microbes, characterized by increased abundance of periodontal pathogens and reduced abundance of probiotics [35]. We employed MaAsLin2 to perform differential abundance analysis comparing the healthy group with the moderate and severe periodontitis groups, and defined periodontitis-associated differential oral microbes as those exhibiting significant differential abundance between patients with both moderate and severe periodontitis and healthy individuals. At the Pla niche, the periodontal pathogens *F.alocis* [26] and *E. brachy* [25] were consistently identified as periodontitis-associated differential oral microbes under both T2DM and non-T2DM conditions. Previous studies have shown that the detection rate of *F.alocis* is significantly higher in subjects with moderately or poorly controlled glycemia compared with those with well-controlled glycemia [36]. Prior research has examined serum immunoglobulin G (IgG) responses to the cell envelope proteins (CEPs) from *C. sputigena* and *C. gingivalis* in periodontally healthy individuals and patients with periodontitis, including those with and without T1DM [37]. *C. sputigena* and *C. gingivalis* were identified as periodontitis-associated differential oral microbes T2DM-specific, and significantly down-regulated in the periodontitis group. These periodontitis-related differential oral microbes identified in this study may play a vital role in periodontitis induced by T2DM.

Key pathogens can promote periodontitis severity by supporting and shaping microbial community structures [38]. The NetMoss method [22] was employed to compare the microbial interaction networks between the healthy group and the T2DM-associated moderate and severe periodontitis groups. It was discovered that the key pathogens that promote periodontitis severity in the ND state are common periodontal pathogens, including *T. forsythia, P. gingivalis*, *F. alocis*, etc. Nevertheless, key pathogens have changed in the T2DM state. *S. leidyi, C. morbi* and *A. massiliensis* propel the transition from T2DM-H to T2DM-MP. *S. moorei, F. hwasookii and C. morbi* drive the transition from T2DM-H to T2DM-SP. Further investigation into the roles of these key pathogens identified here will facilitate a deeper understanding of how T2DM promotes periodontitis. Due to the relatively small sample size of the T2DM-H group (*n* = 9), related analyses may have been limited by insufficient statistical power. This could affect the stability and reliability of MaAsLin2 differential abundance and the NetMoss network analyses. Thus, the potential T2DM-associated network biomarkers identified on the basis of the T2DM-H group should be interpreted as exploratory candidate biomarkers.

Microbial crosstalk across oral niches is pivotal to the severity of periodontitis. As periodontitis severity increases, disease-associated oral microbes that initially colonize other oral niches gradually colonize the GCF, damage periodontal tissues through attachment to pockets or tooth surfaces, and thus accelerate progression [39]. Microbial crosstalk among distinct oral niches at different periodontitis severity in the diabetic state was analyzed. The results from FEAST-based source tracking [23] revealed that microbial source contributions among distinct oral niches underwent significant differences when periodontitis severity increased in the T2DM state. The increased source contribution rates may be attributable to multiple factors such as local inflammation, oral hygiene status, normal physiological microbial flow and disease-associated alterations in the microenvironment. In the meantime, iCAMP [24] analysis in this study indicated that under the T2DM condition, oral microbial community assembly shifts from stochastic to deterministic dominance when periodontitis severity increased. Overall, the iCAMP results should be explained as suggestive evidence, which indicates that disease-associated microenvironmental alterations may cause differences in microbial community assembly processes. Future studies that combine multi-omics approaches with mechanistic investigations are warranted to further elucidate the biological mechanisms underlying these observations.

Periodontal therapy is mainly aimed at reducing or eliminating pathogenic species and maintaining the colonization of host-compatible species [40,41]. However, whether these reduced or eliminated pathogenic bacteria are potential candidate taxa of periodontitis progression under the T2DM condition remains unclear. It was noticed that at the Pla niche, the abundance of *P. gingivalis, T. forsythia* and *E. brachy*, which was identified as a key bacterium driving periodontitis severity increase, decreased significantly after periodontal therapy. However, these pathogenic bacteria were not identified as key biomarkers of periodontitis severity under T2DM conditions in the present study. In contrast, periodontitis pathogen *S. moorei* [42] is one of the 12 microbes. It exhibited significant differences before and after glycemic control and was also identified in the present study as a key bacterium driving periodontitis severity increase under the T2DM condition. These oral microbes may be viewed as potential microbial biomarkers for the treatment of periodontitis with T2DM. In addition, longitudinal follow-up data after treatment were lacking in this study, which limited the ability to evaluate the durability and long-term stability of treatment-associated differentiated oral microbial profiles, as well as their relationships with long-term clinical outcomes. In future studies, longitudinal follow-up samples collected at multiple post-treatment time points should be included to dynamically assess the persistence of post-treatment differentiated oral microbial profiles and clarify their potential associations with disease prognosis.

In the current study, *S. moorei*, *C. morbi* and other taxa were innovatively identified as potential biomarkers of periodontitis severity in T2DM subjects. Their potential pathogenic mechanisms may be in connection with their inherent virulence traits and interactions with the T2DM-related oral microenvironment. As a Gram-positive obligate anaerobic bacillus, *S. moorei* is closely linked to oral malodor and can produce harmful metabolites disrupting oral homeostasis [43]. It has also been isolated from the subgingival Pla of patients with refractory periodontitis [44], which suggests its involvement in periodontal tissue damage. *C. morbi*, a Gram-negative anaerobic bacillus isolated from human gingival crevices, is related to periodontitis and endodontic infections [45,46]. Its elevated abundance is considered a potential biomarker of periodontitis, which may contribute to disease severity through triggering local inflammatory responses. Moreover, the putative virulence factors of these key biomarkers, like metabolic product secretion and immunomodulatory capabilities, may further expedite periodontal inflammation under the T2DM condition despite the necessity of further validating their specific regulatory roles in T2DM-associated periodontitis.

Several limitations should be noted in this study. Firstly, only nine subjects were included in the T2DM-H group (T2DM without periodontitis), which was much smaller than the other seven groups. This unbalanced sample size may have an influence on the stability of the statistical results. Accordingly, the conclusion that specific bacterial species are key biomarkers of periodontitis progression under the T2DM condition should be interpreted cautiously owing to the limited sample size in this group. Future studies with larger and more balanced sample sizes are required to verify these observations.

## Conclusion

This study confirms that T2DM largely reshapes periodontitis-associated oral microbial dysbiosis patterns, which additionally display T2DM-specific microbial traits. This highlights the unique regulatory role and significant impact of T2DM on oral microbiome alterations in periodontitis. During periodontitis progression under the T2DM condition, a significant reduction was observed in correlations between differential microbes and clinical parameters. *S. leidyi*, *C. morbi*, *A. massiliensis*, *S. moorei*, etc., were further identified as potential pathogenic biomarkers of periodontitis progression. In addition, T2DM may also accelerate periodontitis progression via the disruption of microbial crosstalk among distinct oral niches.

## Figures and Tables

**Figure A1.**
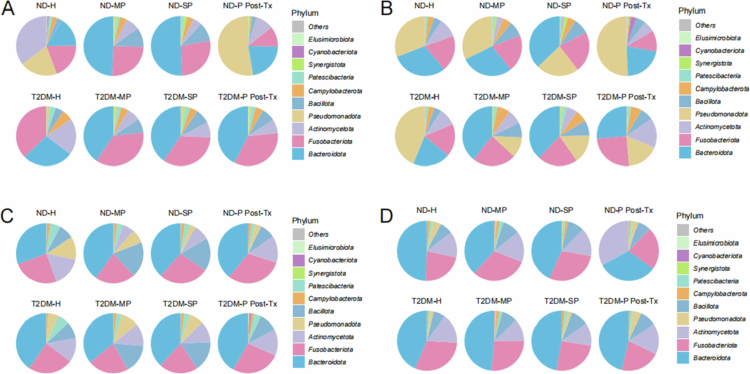
The relative abundances of the top 10 bacterial phyla in all individual samples from ND and T2DM subjects. A–D. Relative abundance profiles of the top 10 bacterial phyla at the Pla/GCF/Sal/TB niche across the different groups.

**Figure A2.**
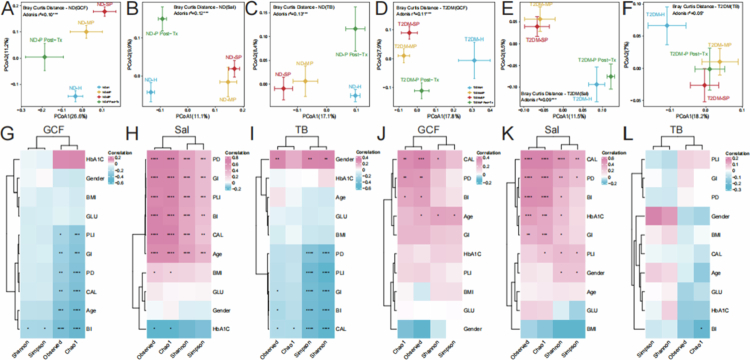
Microbial community diversity analysis of the GCF, Sal, and TB niches. A. PCoA based on Bray-Curtis distance showing community differences among the ND-H, ND-MP, ND-SP, and ND-P Post-tx groups at the GCF niche. B. PCoA based on Bray-Curtis distance showing community differences among the T2DM-H, T2DM-MP, T2DM-SP, and T2DM-P Post-tx groups at the GCF niche. C. PCoA based on Bray-Curtis distance showing community differences among the ND-H, ND-MP, ND-SP, and ND-P Post-tx groups at the Sal niche. D. PCoA based on Bray-Curtis distance showing community differences among the T2DM-H, T2DM-MP, T2DM-SP, and T2DM-P Post-tx groups at the Sal niche. E. PCoA based on Bray–Curtis distance showing community differences among the ND-H, ND-MP, ND-SP, and ND-P Post-tx groups at the TB niche. F. PCoA based on Bray–Curtis distance showing community differences among the T2DM-H, T2DM-MP, T2DM-SP, and T2DM-P Post-tx groups at the TB niche. G–I. The correlations between clinical parameters and alpha diversity indices of the GCF/Sal/TB niche in ND subjects. J–L. The correlations between clinical parameters and alpha diversity indices of the GCF/Sal/TB niche in T2DM subjects. *p-value < 0.05, **p-value < 0.01, ***p-value < 0.001, ****p-value < 0.0001.

**Figure A3.**
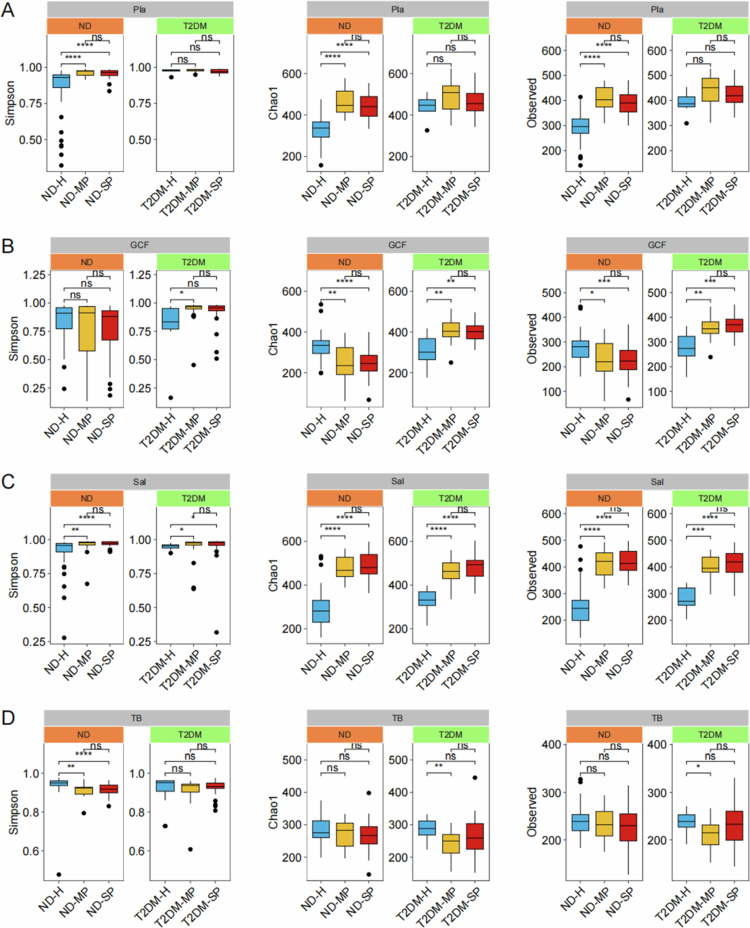
Alpha diversity analysis. A–D. Boxplots showing the distribution and intergroup comparisons of Simpson, Chao1, Observed diversity among healthy, moderate periodontitis, and severe periodontitis groups in ND and T2DM subjects. *p-value < 0.05, **p-value < 0.01, ***p-value < 0.001, ****p-value < 0.0001.

**Figure A4.**
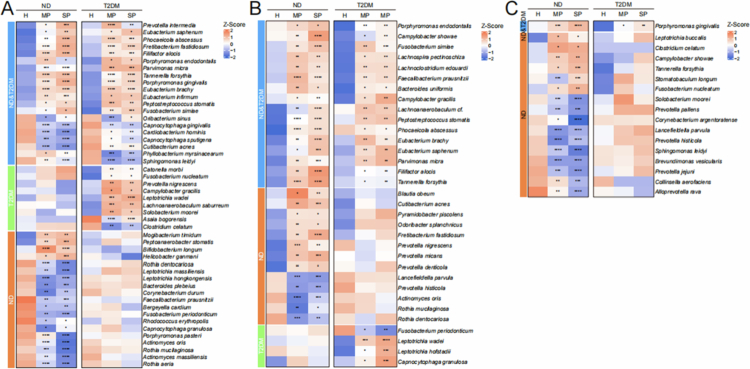
Heatmap of microbes showing significant differences between the periodontitis groups and the healthy group. A–C. Heatmaps of microbial taxa significant differences between moderate or severe periodontitis and healthy groups in the GCF/Sal/TB niche of ND and T2DM subjects. Microbes were classified according to the following criteria: taxa showing significant differences only in ND subjects, only in T2DM subjects, and in both ND and T2DM subjects. *p-value < 0.05, **p-value < 0.01, ***p-value < 0.001, ****p-value < 0.0001.

**Figure A5.**
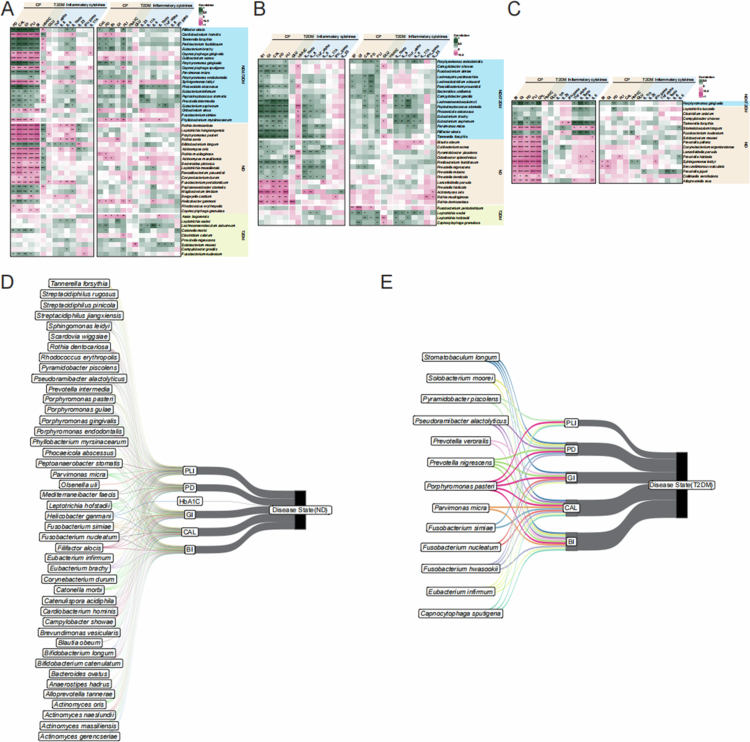
Correlation analysis between significantly different microbes and clinical parameters. A–C. Results of correlation analysis between clinical parameters of ND and T2DM subjects and differential species in the GCF/Sal/TB niche, respectively. D. Mediation analysis of the associations among significantly different microbes, clinical parameters, and periodontitis progression at the Pla niche in ND subjects. E. Mediation analysis of the associations among significantly different microbes, clinical parameters, and periodontitis progression at the Pla niche in T2DM subjects. *p-value < 0.05, **p-value < 0.01, ***p-value < 0.001, ****p-value < 0.0001.

**Figure A6.**
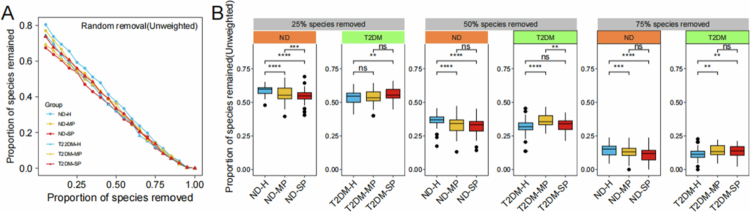
The robustness values of each microbial co-occurrence network. A. Line plots showing the robustness values (Unweighted) of each microbial co-occurrence network. B. Comparison of network robustness (Unweighted) among groups after removing 25%, 50%, and 75% of nodes from the co-occurrence networks. *p-value < 0.05, **p-value < 0.01, ***p-value < 0.001, ****p-value < 0.0001.

**Figure A7.**
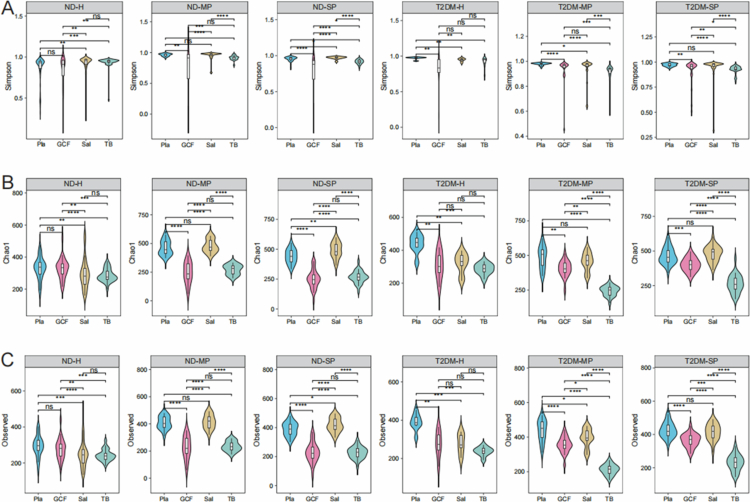
The distribution of Alpha diversity across oral ecological niches under different periodontitis and T2DM conditions. A–C. Violin plots showing the distribution of Simpson/Chao1/Observed diversity across oral ecological niches under different periodontitis and T2DM statuses. *p-value < 0.05, **p-value < 0.01, ***p-value < 0.001, ****p-value < 0.0001.

**Figure A8.**
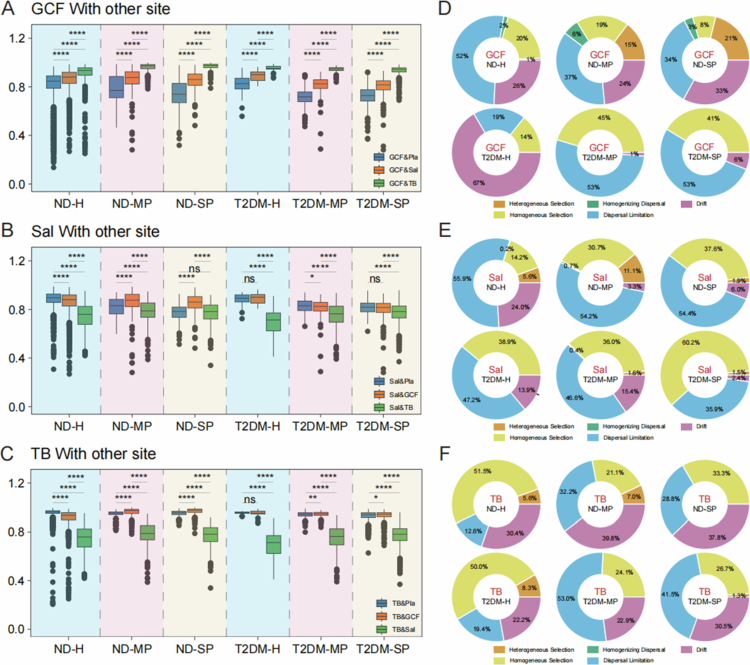
The community assembly mechanisms across oral ecological niches under different periodontitis and T2DM conditions. A. Bray-Curtis distances between the GCF niche and other oral ecological niches across different periodontitis and T2DM statuses. B. Bray-Curtis distances between the Sal niche and other oral ecological niches across different periodontitis and T2DM statuses. C. Bray-Curtis distances beween the TB niche and other oral ecological niches across different periodontitis and T2DM statuses. D–E. Contributions of deterministic and stochastic processes to community assembly mechanisms in the GCF/Sal/TB niche under different periodontitis and T2DM statuses. *p-value < 0.05, **p-value < 0.01, ****p-value < 0.0001.

**Figure A9.**
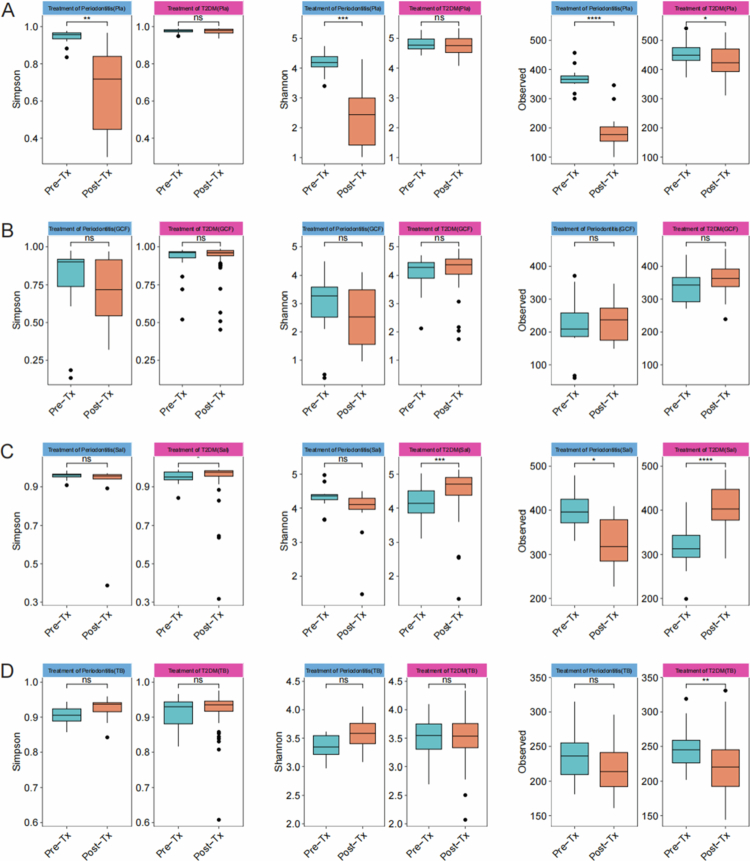
The distribution of Alaph diversity indices before and after periodontal treatment, as well as before and after T2DM treatment. A–D. Boxplots illustrating the distribution of Simpson, Shannon, Observed diversity indices in the Pla/GCF/Sal/TB niche before and after periodontal treatment and T2DM treatment.

**Figure A10.**
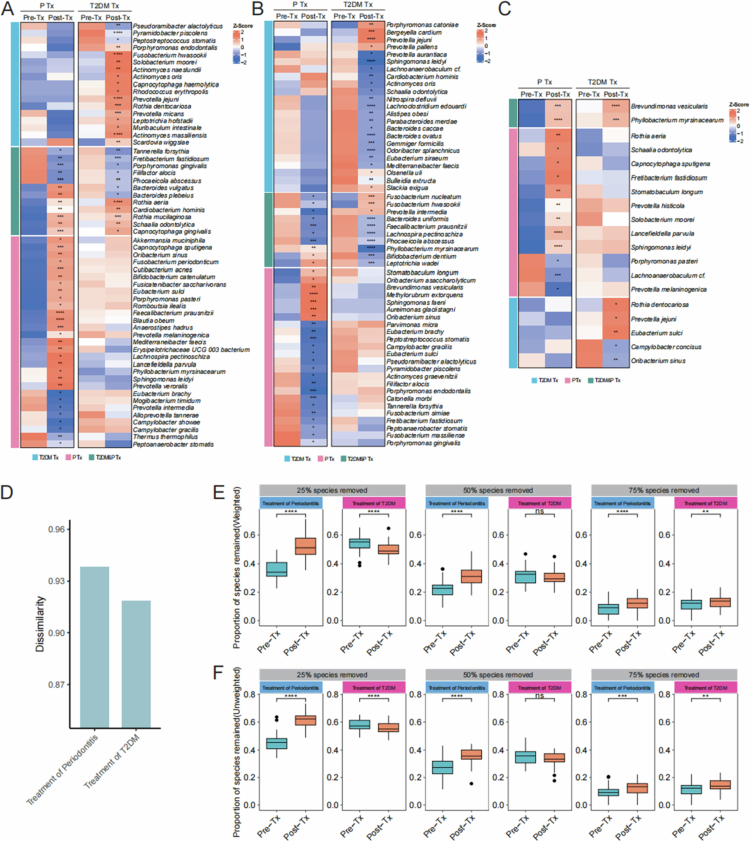
Impacts of periodontal and T2DM treatments on microbial community changes and network characteristics across oral ecological niches. A–C. Heatmaps of microbial taxa significant differences between before and after treatment in the GCF/Sal/TB niche of periodontal treatment and T2DM treatment. Microbes were classified according to the following criteria: taxa showing significant differences only in periodontal treatment, only in T2DM treatment., and in both periodontal treatment and T2DM treatment. D. Network dissimilarity index of periodontal and T2DM treatments. E. Comparison of network robustness (Weighted) among groups after removing 25%, 50%, and 75% of nodes from the co-occurrence networks. F. Comparison of network robustness (Unweighted) among groups after removing 25%, 50%, and 75% of nodes from the co-occurrence networks.

## Data Availability

The raw sequence data used in this paper were deposited in the Genome Sequence Archive (Genomics, Proteomics & Bioinformatics 2021) in the National Genomics Data Center (Nucleic Acids Res 2022), China National Center for Bioinformation/Beijing Institute of Genomics, Chinese Academy of Sciences (accession number GSA: CRA022445), and are publicly accessible at https://ngdc.cncb.ac.cn/gsa.

## Appendices

### Appendix Table 1

**Table A1.** Clinical characteristics of the study participants.

	ND-H	ND-MP	ND-SP	T2DM	T2DM-MP	T2DM-SP	T2DM-Post Tx	Kruskal–Wallis pvalue
**Age**	24.82 ± 7.02	44.58 ± 14.37	44.55 ± 12.04	44.67 ± 10.75	60.43 ± 9.67	59.55 ± 12.52	59.00 ± 8.18	7.35E-26
**Gender**								3.05E-01
male	17(34%)	7(37%)	12(39%)	3(33%)	5(22%)	16(52%)	13(59%)	
female	33(66%)	12(63%)	19(61%)	6(67%)	18(78%)	15(48%)	9(41%)	
**BMI**	21.26 ± 2.90	24.19 ± 3.68	22.95 ± 3.15	27.59 ± 1.80	25.24 ± 4.01	25.60 ± 3.99	25.39 ± 3.39	1.98E-09
**HbA1C**	4.99 ± 0.49	4.69 ± 0.58	4.78 ± 0.53	8.43 ± 1.41	8.95 ± 0.83	9.14 ± 1.78	5.43 ± 0.63	3.13E-29
**GLU**	5.28 ± 0.38	5.35 ± 0.40	5.31 ± 0.56	11.66 ± 1.87	9.74 ± 2.21	9.72 ± 2.56	6.38 ± 0.67	3.92E-33
**BI**	0.66 ± 0.52	3.06 ± 0.64	3.23 ± 0.50	1.11 ± 0.33	3.05 ± 0.65	3.34 ± 0.48	2.62 ± 0.50	7.84E-29
**PLI**	1.06 ± 0.31	2.39 ± 0.61	2.90 ± 0.70	1.11 ± 0.33	2.14 ± 0.71	2.41 ± 0.50	2.52 ± 0.51	6.73E-25
**GI**	0.50 ± 0.51	2.33 ± 0.49	2.84 ± 0.52	1.00 ± 0.5	1.64 ± 0.73	2.41 ± 0.57	2.14 ± 0.57	1.71E-26
**PD**	1.92 ± 0.49	5.44 ± 0.92	7.19 ± 1.76	2.11 ± 0.78	5.23 ± 1.15	6.66 ± 1.29	5.29 ± 1.38	1.61E-28
**CAL**	0.00 + 0.00	3.61 ± 1.20	5.48 ± 1.81	0.00 + 0.00	3.68 ± 0.95	5.10 ± 1.08	3.57 ± 1.25	4.64E-29

### Appendix Table 2

**Table A2.** Species significantly differing between the moderate/severe periodontitis groups and the healthy group across different oral niches.

Feature	Value	Coef	Pval
*Species significantly differing between the moderate/severe periodontitis groups and the healthy group in the Pla niche*			
*Phocaeicola abscessus*	ND-MP vs. ND-H	*6.659676141*	1.39203E-22
*Eubacterium brachy*	ND-MP vs. ND-H	*5.166569422*	1.10132E-21
*Tannerella forsythia*	ND-MP vs. ND-H	*5.726805615*	2.2837E-19
*Porphyromonas gingivalis*	ND-MP vs. ND-H	*7.321588822*	1.73733E-18
*Filifactor alocis*	ND-MP vs. ND-H	*4.933006719*	2.17871E-18
*Peptoanaerobacter stomatis*	ND-MP vs. ND-H	*3.335582364*	6.04362E-14
*Prevotella denticola*	ND-MP vs. ND-H	*4.518177531*	1.068E-13
*Alloprevotella tannerae*	ND-MP vs. ND-H	*4.104477452*	1.65374E-12
*Parvimonas micra*	ND-MP vs. ND-H	*3.59512338*	3.65415E-11
*Prevotella veroralis*	ND-MP vs. ND-H	*4.056795402*	5.95618E-10
*Prevotella nigrescens*	ND-MP vs. ND-H	*3.634574271*	3.05963E-09
*Stomatobaculum longum*	ND-MP vs. ND-H	*3.287715933*	5.06064E-08
*Eubacterium infirmum*	ND-MP vs. ND-H	*2.935200057*	1.4744E-07
*Solobacterium moorei*	ND-MP vs. ND-H	*2.522842888*	1.82364E-06
*Rothia dentocariosa*	ND-MP vs. ND-H	*−3.517499179*	3.39116E-06
*Pseudoramibacter alactolyticus*	ND-MP vs. ND-H	*1.815784771*	1.63746E-05
*Actinomyces naeslundii*	ND-MP vs. ND-H	*−3.072321262*	9.55241E-05
*Porphyromonas pasteri*	ND-MP vs. ND-H	*−2.330832547*	0.000287976
*Actinomyces oris*	ND-MP vs. ND-H	*−2.575996964*	0.000364064
*Corynebacterium durum*	ND-MP vs. ND-H	*−3.071582539*	0.000465824
*Porphyromonas endodontalis*	ND-MP vs. ND-H	*5.941834543*	5.92589E-17
*Olsenella uli*	ND-MP vs. ND-H	*2.60301683*	8.02208E-11
*Fusobacterium nucleatum*	ND-MP vs. ND-H	*1.683378195*	1.11311E-09
*Actinomyces massiliensis*	ND-MP vs. ND-H	*−3.784667566*	2.03585E-09
*Fusobacterium simiae*	ND-MP vs. ND-H	*3.395368006*	6.31278E-08
*Cardiobacterium hominis*	ND-MP vs. ND-H	*−2.430443099*	3.7436E-07
*Catonella morbi*	ND-MP vs. ND-H	*1.888131003*	2.3646E-06
*Prevotella intermedia*	ND-MP vs. ND-H	*4.114997605*	4.00196E-06
*Leptotrichia hofstadii*	ND-MP vs. ND-H	*−2.157583362*	9.79437E-06
*Phyllobacterium myrsinacearum*	ND-MP vs. ND-H	*−5.063020135*	2.47137E-05
*Pyramidobacter piscolens*	ND-MP vs. ND-H	*1.480443922*	0.000273369
*Blautia obeum*	ND-MP vs. ND-H	*−2.531261007*	0.00044946
*Rhodococcus erythropolis*	ND-MP vs. ND-H	*−1.248325283*	0.000490088
*Sphingomonas leidyi*	ND-MP vs. ND-H	*−2.863127721*	0.00053075
*Prevotella melaninogenica*	ND-MP vs. ND-H	*2.373936352*	0.000748554
*Actinomyces gerencseriae*	ND-MP vs. ND-H	*−2.29590122*	0.00081686
*Scardovia wiggsiae*	ND-MP vs. ND-H	*1.382256396*	0.000944108
*Porphyromonas gulae*	ND-MP vs. ND-H	*1.709328807*	0.00119728
*Mediterraneibacter faecis*	ND-MP vs. ND-H	*−1.077321281*	0.002013073
*Anaerostipes hadrus*	ND-MP vs. ND-H	*−1.279620628*	0.002058454
*Faecalibacterium prausnitzii*	ND-MP vs. ND-H	*−1.77383057*	0.006023311
*Streptacidiphilus pinicola*	ND-MP vs. ND-H	*−0.833173223*	0.007657409
*Campylobacter showae*	ND-MP vs. ND-H	*1.143335189*	0.00942196
*Bifidobacterium longum*	ND-MP vs. ND-H	*−1.485940773*	0.010596726
*Streptacidiphilus jiangxiensis*	ND-MP vs. ND-H	*−0.86602699*	0.011248622
*Catenulispora acidiphila*	ND-MP vs. ND-H	*−0.566189108*	0.01257485
*Brevundimonas vesicularis*	ND-MP vs. ND-H	*−1.004078594*	0.015930361
*Bacteroides ovatus*	ND-MP vs. ND-H	*−1.091620855*	0.01938982
*Prevotella micans*	ND-MP vs. ND-H	*1.875206937*	0.026074663
*Bacteroides vulgatus*	ND-MP vs. ND-H	*−1.16709963*	0.027625644
*Helicobacter ganmani*	ND-MP vs. ND-H	*−1.338250219*	0.030421554
*Bifidobacterium catenulatum*	ND-MP vs. ND-H	*−1.402887595*	0.039457764
*Streptacidiphilus rugosus*	ND-MP vs. ND-H	*−0.58008766*	0.046228607
*Phocaeicola abscessus*	ND-SP vs. ND-H	*6.530759093*	1.59284E-24
*Eubacterium brachy*	ND-SP vs. ND-H	*5.008372938*	2.81486E-23
*Tannerella forsythia*	ND-SP vs. ND-H	*6.193322449*	1.19451E-23
*Porphyromonas gingivalis*	ND-SP vs. ND-H	*7.707425134*	5.84489E-22
*Filifactor alocis*	ND-SP vs. ND-H	*5.160618003*	1.08214E-21
*Peptoanaerobacter stomatis*	ND-SP vs. ND-H	*3.254721361*	2.95523E-15
*Prevotella denticola*	ND-SP vs. ND-H	*3.637658512*	1.37252E-11
*Alloprevotella tannerae*	ND-SP vs. ND-H	*3.620095322*	5.74977E-12
*Parvimonas micra*	ND-SP vs. ND-H	*3.382712181*	1.14691E-11
*Prevotella veroralis*	ND-SP vs. ND-H	*3.362953303*	1.02481E-08
*Prevotella nigrescens*	ND-SP vs. ND-H	*3.397408918*	1.4091E-09
*Stomatobaculum longum*	ND-SP vs. ND-H	*2.328725165*	1.24806E-05
*Eubacterium infirmum*	ND-SP vs. ND-H	*2.64695009*	1.89798E-07
*Solobacterium moorei*	ND-SP vs. ND-H	*2.32983193*	1.37304E-06
*Rothia dentocariosa*	ND-SP vs. ND-H	*−4.027348013*	1.50339E-08
*Pseudoramibacter alactolyticus*	ND-SP vs. ND-H	*2.074936745*	1.41911E-07
*Actinomyces naeslundii*	ND-SP vs. ND-H	*−3.161191181*	1.31074E-05
*Porphyromonas pasteri*	ND-SP vs. ND-H	*−1.817675311*	0.001731151
*Actinomyces oris*	ND-SP vs. ND-H	*−2.618321525*	8.00277E-05
*Corynebacterium durum*	ND-SP vs. ND-H	*−3.841355642*	2.97348E-06
*Porphyromonas endodontalis*	ND-SP vs. ND-H	*5.637156729*	7.82188E-18
*Olsenella uli*	ND-SP vs. ND-H	*2.02248547*	1.15964E-08
*Fusobacterium nucleatum*	ND-SP vs. ND-H	*1.938930841*	2.383E-13
*Actinomyces massiliensis*	ND-SP vs. ND-H	*−4.0457197*	9.4405E-12
*Fusobacterium simiae*	ND-SP vs. ND-H	*3.677233388*	4.19723E-10
*Cardiobacterium hominis*	ND-SP vs. ND-H	*−2.380918935*	6.35276E-08
*Catonella morbi*	ND-SP vs. ND-H	*2.220204087*	4.22082E-09
*Prevotella intermedia*	ND-SP vs. ND-H	*3.66617674*	6.19134E-06
*Leptotrichia hofstadii*	ND-SP vs. ND-H	*−2.776891765*	2.52481E-09
*Phyllobacterium myrsinacearum*	ND-SP vs. ND-H	*−4.797156712*	1.24806E-05
*Pyramidobacter piscolens*	ND-SP vs. ND-H	*2.082687488*	7.85501E-08
*Blautia obeum*	ND-SP vs. ND-H	*−2.489246353*	0.000165164
*Rhodococcus erythropolis*	ND-SP vs. ND-H	*−1.280808866*	9.94174E-05
*Sphingomonas leidyi*	ND-SP vs. ND-H	*−2.666007444*	0.000406737
*Prevotella melaninogenica*	ND-SP vs. ND-H	*2.464247188*	0.000141011
*Actinomyces gerencseriae*	ND-SP vs. ND-H	*−2.698123984*	2.29181E-05
*Scardovia wiggsiae*	ND-SP vs. ND-H	*1.538181019*	6.83569E-05
*Porphyromonas gulae*	ND-SP vs. ND-H	*1.712802009*	0.000397423
*Mediterraneibacter faecis*	ND-SP vs. ND-H	*−1.119417516*	0.000473994
*Anaerostipes hadrus*	ND-SP vs. ND-H	*−1.302296427*	0.000625081
*Faecalibacterium prausnitzii*	ND-SP vs. ND-H	*−2.221424*	0.000208331
*Streptacidiphilus pinicola*	ND-SP vs. ND-H	*−0.851293546*	0.00292316
*Campylobacter showae*	ND-SP vs. ND-H	*1.220452218*	0.002518136
*Bifidobacterium longum*	ND-SP vs. ND-H	*−1.398796645*	0.008359143
*Streptacidiphilus jiangxiensis*	ND-SP vs. ND-H	*−0.877529928*	0.004995057
*Catenulispora acidiphila*	ND-SP vs. ND-H	*−0.566670911*	0.006301857
*Brevundimonas vesicularis*	ND-SP vs. ND-H	*−0.893893347*	0.018429512
*Bacteroides ovatus*	ND-SP vs. ND-H	*−1.111903758*	0.009218178
*Prevotella micans*	ND-SP vs. ND-H	*2.028721178*	0.00860388
*Bacteroides vulgatus*	ND-SP vs. ND-H	*−1.104018945*	0.022356399
*Helicobacter ganmani*	ND-SP vs. ND-H	*−1.314598622*	0.019873298
*Bifidobacterium catenulatum*	ND-SP vs. ND-H	*−1.295023465*	0.037004497
*Streptacidiphilus rugosus*	ND-SP vs. ND-H	*−0.567758226*	0.032555214
*Phocaeicola abscessus*	T2DM-MP vs. T2DM-H	*1.988756903*	0.009073278
*Eubacterium brachy*	T2DM-MP vs. T2DM-H	*2.532631481*	9.38207E-05
*Tannerella forsythia*	T2DM-MP vs. T2DM-H	*2.361767858*	0.00089753
*Porphyromonas gingivalis*	T2DM-MP vs. T2DM-H	*3.806249645*	2.07318E-05
*Filifactor alocis*	T2DM-MP vs. T2DM-H	*1.951786447*	0.001497059
*Peptoanaerobacter stomatis*	T2DM-MP vs. T2DM-H	*2.836862259*	0.000169751
*Prevotella denticola*	T2DM-MP vs. T2DM-H	*4.848682623*	4.23488E-05
*Alloprevotella tannerae*	T2DM-MP vs. T2DM-H	*2.948324081*	0.003343336
*Parvimonas micra*	T2DM-MP vs. T2DM-H	*1.447456984*	0.003922627
*Prevotella veroralis*	T2DM-MP vs. T2DM-H	*2.132234788*	0.013640342
*Prevotella nigrescens*	T2DM-MP vs. T2DM-H	*2.506394841*	6.92119E-06
*Stomatobaculum longum*	T2DM-MP vs. T2DM-H	*1.785762324*	0.002967173
*Eubacterium infirmum*	T2DM-MP vs. T2DM-H	*2.433767729*	0.000425132
*Solobacterium moorei*	T2DM-MP vs. T2DM-H	*2.512226191*	5.6643E-06
*Rothia dentocariosa*	T2DM-MP vs. T2DM-H	*−3.158344549*	0.003315775
*Pseudoramibacter alactolyticus*	T2DM-MP vs. T2DM-H	*2.077043563*	0.008194122
*Actinomyces naeslundii*	T2DM-MP vs. T2DM-H	*−3.01583118*	0.003093647
*Porphyromonas pasteri*	T2DM-MP vs. T2DM-H	*−1.707606042*	0.004738118
*Actinomyces oris*	T2DM-MP vs. T2DM-H	*−4.545042886*	5.05198E-06
*Corynebacterium durum*	T2DM-MP vs. T2DM-H	*−3.144200813*	0.005565261
*Capnocytophaga gingivalis*	T2DM-MP vs. T2DM-H	*−2.20952544*	0.003150763
*Capnocytophaga sputigena*	T2DM-MP vs. T2DM-H	*−1.972054626*	0.004627866
*Fusobacterium hwasookii*	T2DM-MP vs. T2DM-H	*−1.683154735*	0.007662704
*Phocaeicola abscessus*	T2DM-SP vs. T2DM-H	*2.009029911*	0.009581276
*Eubacterium brachy*	T2DM-SP vs. T2DM-H	*2.305108468*	0.000397643
*Tannerella forsythia*	T2DM-SP vs. T2DM-H	*2.066691232*	0.003809746
*Porphyromonas gingivalis*	T2DM-SP vs. T2DM-H	*3.78800633*	2.96151E-05
*Filifactor alocis*	T2DM-SP vs. T2DM-H	*1.4393895*	0.018579699
*Peptoanaerobacter stomatis*	T2DM-SP vs. T2DM-H	*3.310270675*	2.36416E-05
*Prevotella denticola*	T2DM-SP vs. T2DM-H	*4.559667937*	0.000131073
*Alloprevotella tannerae*	T2DM-SP vs. T2DM-H	*3.197628966*	0.001874849
*Parvimonas micra*	T2DM-SP vs. T2DM-H	*1.589205198*	0.001992683
*Prevotella veroralis*	T2DM-SP vs. T2DM-H	*2.407481299*	0.006570464
*Prevotella nigrescens*	T2DM-SP vs. T2DM-H	*2.566745563*	6.23502E-06
*Stomatobaculum longum*	T2DM-SP vs. T2DM-H	*2.29935329*	0.00024276
*Eubacterium infirmum*	T2DM-SP vs. T2DM-H	*2.367610044*	0.000713503
*Solobacterium moorei*	T2DM-SP vs. T2DM-H	*2.630725753*	3.40584E-06
*Rothia dentocariosa*	T2DM-SP vs. T2DM-H	*−2.416952492*	0.024579786
*Pseudoramibacter alactolyticus*	T2DM-SP vs. T2DM-H	*2.549286409*	0.001680759
*Actinomyces naeslundii*	T2DM-SP vs. T2DM-H	*−2.113382657*	0.037312766
*Porphyromonas pasteri*	T2DM-SP vs. T2DM-H	*−2.216779789*	0.000429484
*Actinomyces oris*	T2DM-SP vs. T2DM-H	*−3.771570045*	0.000123363
*Corynebacterium durum*	T2DM-SP vs. T2DM-H	*−3.144913471*	0.006383435
*Capnocytophaga gingivalis*	T2DM-SP vs. T2DM-H	*−2.365460262*	0.001988837
*Capnocytophaga sputigena*	T2DM-SP vs. T2DM-H	*−2.295227542*	0.001368598
*Fusobacterium hwasookii*	T2DM-SP vs. T2DM-H	*−2.328567341*	0.000420641
Feature	Value	Coef	Pval
*Species significantly differing between the moderate/severe periodontitis groups and the healthy group in the TB niche*			
*Porphyromonas gingivalis*	*ND-MP vs. ND-H*	*2.291174795*	*0.000834573*
*Sphingomonas leidyi*	*ND-MP vs. ND-H*	*−4.194747232*	*5.84172E-12*
*Prevotella histicola*	*ND-MP vs. ND-H*	*−4.371313602*	*2.44675E-11*
*Lancefieldella parvula*	*ND-MP vs. ND-H*	*−1.969401838*	*1.57035E-06*
*Brevundimonas vesicularis*	*ND-MP vs. ND-H*	*−2.867432069*	*4.81662E-06*
*Solobacterium moorei*	*ND-MP vs. ND-H*	*−1.096651704*	*0.000316509*
*Prevotella jejuni*	*ND-MP vs. ND-H*	*−2.674099423*	*0.000496561*
*Stomatobaculum longum*	*ND-MP vs. ND-H*	*−2.118387293*	*0.000638202*
*Prevotella pallens*	*ND-MP vs. ND-H*	*−2.17717871*	*0.000931673*
*Alloprevotella rava*	*ND-MP vs. ND-H*	*−2.380946033*	*0.005306304*
*Campylobacter showae*	*ND-MP vs. ND-H*	*1.342795058*	*0.007111553*
*Collinsella aerofaciens*	*ND-MP vs. ND-H*	*−1.189693831*	*0.008932449*
*Corynebacterium argentoratense*	*ND-MP vs. ND-H*	*−1.07875484*	*0.01355921*
*Leptotrichia buccalis*	*ND-MP vs. ND-H*	*1.111810322*	*0.019271325*
*Clostridium celatum*	*ND-MP vs. ND-H*	*1.159284606*	*0.019965716*
*Tannerella forsythia*	*ND-MP vs. ND-H*	*1.508853549*	*0.020198841*
*Fusobacterium nucleatum*	*ND-MP vs. ND-H*	*1.013861524*	*0.030908337*
*Porphyromonas gingivalis*	*ND-SP vs. ND-H*	*2.995177861*	*3.29926E-06*
*Sphingomonas leidyi*	*ND-SP vs. ND-H*	*−4.19421718*	*1.47985E-13*
*Prevotella histicola*	*ND-SP vs. ND-H*	*−4.506927361*	*2.09051E-13*
*Lancefieldella parvula*	*ND-SP vs. ND-H*	*−2.094186411*	*4.03637E-08*
*Brevundimonas vesicularis*	*ND-SP vs. ND-H*	*−2.738306501*	*1.8684E-06*
*Solobacterium moorei*	*ND-SP vs. ND-H*	*−1.381751299*	*1.34495E-06*
*Prevotella jejuni*	*ND-SP vs. ND-H*	*−3.690900757*	*3.93228E-07*
*Stomatobaculum longum*	*ND-SP vs. ND-H*	*−2.200434882*	*0.000114681*
*Prevotella pallens*	*ND-SP vs. ND-H*	*−2.321837463*	*0.000126413*
*Alloprevotella rava*	*ND-SP vs. ND-H*	*−3.376658692*	*2.47805E-05*
*Campylobacter showae*	*ND-SP vs. ND-H*	*1.201320636*	*0.00817872*
*Collinsella aerofaciens*	*ND-SP vs. ND-H*	*−1.397306608*	*0.000868923*
*Corynebacterium argentoratense*	*ND-SP vs. ND-H*	*−1.687589813*	*3.95533E-05*
*Leptotrichia buccalis*	*ND-SP vs. ND-H*	*1.052688819*	*0.015144921*
*Clostridium celatum*	*ND-SP vs. ND-H*	*0.913719966*	*0.043338684*
*Tannerella forsythia*	*ND-SP vs. ND-H*	*2.135698239*	*0.000406238*
*Fusobacterium nucleatum*	*ND-SP vs. ND-H*	*1.267729596*	*0.003399356*
*Porphyromonas gingivalis*	*T2DM-MP vs. T2DM-H*	*1.958260506*	*0.031727936*
*Porphyromonas gingivalis*	*T2DM-SP vs. T2DM-H*	*2.550112991*	*0.00697057*
Feature	Value	Coef	Pval
*Species significantly differing between the moderate/severe periodontitis groups and the healthy group in the GCF niche*			
*Filifactor alocis*	ND-MP vs. ND-H	3.832346357	2.0848E-07
*Cardiobacterium hominis*	ND-MP vs. ND-H	−3.576254915	4.55399E-07
*Tannerella forsythia*	ND-MP vs. ND-H	4.051172663	7.81081E-06
*Fretibacterium fastidiosum*	ND-MP vs. ND-H	3.224432806	2.90136E-05
*Eubacterium brachy*	ND-MP vs. ND-H	3.206163767	3.35508E-05
*Capnocytophaga gingivalis*	ND-MP vs. ND-H	−3.521483353	3.76087E-05
*Cutibacterium acnes*	ND-MP vs. ND-H	−2.095319894	6.17654E-05
*Porphyromonas gingivalis*	ND-MP vs. ND-H	4.068079801	8.9004E-05
*Capnocytophaga sputigena*	ND-MP vs. ND-H	−3.465391376	0.000227561
*Parvimonas micra*	ND-MP vs. ND-H	2.466509764	0.000403785
*Porphyromonas endodontalis*	ND-MP vs. ND-H	3.122566433	0.001388823
*Sphingomonas leidyi*	ND-MP vs. ND-H	−2.966696493	0.001460881
*Phocaeicola abscessus*	ND-MP vs. ND-H	2.984651764	0.001776356
*Eubacterium infirmum*	ND-MP vs. ND-H	1.827333393	0.003460378
*Peptostreptococcus stomatis*	ND-MP vs. ND-H	1.635270651	0.022767195
*Prevotella intermedia*	ND-MP vs. ND-H	2.324828979	0.022748476
*Eubacterium saphenum*	ND-MP vs. ND-H	2.15839436	0.023350864
*Oribacterium sinus*	ND-MP vs. ND-H	−1.032823082	0.027892619
*Fusobacterium simiae*	ND-MP vs. ND-H	2.162175628	0.038630361
*Phyllobacterium myrsinacearum*	ND-MP vs. ND-H	−1.764556653	0.046811355
*Rothia dentocariosa*	ND-MP vs. ND-H	−5.621404454	2.53326E-10
*Leptotrichia hongkongensis*	ND-MP vs. ND-H	−5.615993094	2.27818E-09
*Porphyromonas pasteri*	ND-MP vs. ND-H	−5.640184044	3.67559E-09
*Rothia aeria*	ND-MP vs. ND-H	−4.020894298	5.72183E-07
*Bifidobacterium longum*	ND-MP vs. ND-H	4.760029368	8.52696E-06
*Actinomyces oris*	ND-MP vs. ND-H	−3.08354556	2.42597E-05
*Rothia mucilaginosa*	ND-MP vs. ND-H	−2.323903782	0.000305117
*Actinomyces massiliensis*	ND-MP vs. ND-H	−1.928153298	0.001182457
*Bacteroides plebeius*	ND-MP vs. ND-H	−1.757084955	0.001439899
*Leptotrichia massiliensis*	ND-MP vs. ND-H	−1.76267129	0.002094876
*Faecalibacterium prausnitzii*	ND-MP vs. ND-H	−3.129443753	0.003012455
*Corynebacterium durum*	ND-MP vs. ND-H	−2.060306494	0.003374436
*Fusobacterium periodonticum*	ND-MP vs. ND-H	−2.189434504	0.004345224
*Peptoanaerobacter stomatis*	ND-MP vs. ND-H	1.631849918	0.004937953
*Mogibacterium timidum*	ND-MP vs. ND-H	1.121288413	0.006541003
*Bergeyella cardium*	ND-MP vs. ND-H	−1.482656171	0.014151879
*Helicobacter ganmani*	ND-MP vs. ND-H	1.988549684	0.024632957
*Rhodococcus erythropolis*	ND-MP vs. ND-H	−1.337416975	0.037208217
*Capnocytophaga granulosa*	ND-MP vs. ND-H	−1.621577868	0.043000846
*Filifactor alocis*	ND-SP vs. ND-H	4.419887819	3.7839E-11
*Cardiobacterium hominis*	ND-SP vs. ND-H	−4.321312515	2.36281E-11
*Tannerella forsythia*	ND-SP vs. ND-H	4.322894451	6.85804E-08
*Fretibacterium fastidiosum*	ND-SP vs. ND-H	3.788248083	3.8802E-08
*Eubacterium brachy*	ND-SP vs. ND-H	2.897824591	1.4473E-05
*Capnocytophaga gingivalis*	ND-SP vs. ND-H	−4.534031506	4.73432E-09
*Cutibacterium acnes*	ND-SP vs. ND-H	−2.109987676	3.91207E-06
*Porphyromonas gingivalis*	ND-SP vs. ND-H	5.198837569	2.74111E-08
*Capnocytophaga sputigena*	ND-SP vs. ND-H	−3.945954753	2.02751E-06
*Parvimonas micra*	ND-SP vs. ND-H	2.365837656	9.17134E-05
*Porphyromonas endodontalis*	ND-SP vs. ND-H	1.669023216	0.043227128
*Sphingomonas leidyi*	ND-SP vs. ND-H	−3.828240085	3.87393E-06
*Phocaeicola abscessus*	ND-SP vs. ND-H	4.822826372	3.39077E-08
*Eubacterium infirmum*	ND-SP vs. ND-H	2.004000321	0.000239742
*Peptostreptococcus stomatis*	ND-SP vs. ND-H	1.366034795	0.026912666
*Prevotella intermedia*	ND-SP vs. ND-H	3.00080545	0.000787882
*Eubacterium saphenum*	ND-SP vs. ND-H	2.670939542	0.001314817
*Oribacterium sinus*	ND-SP vs. ND-H	−0.908278296	0.024708775
*Fusobacterium simiae*	ND-SP vs. ND-H	1.976004118	0.028291449
*Phyllobacterium myrsinacearum*	ND-SP vs. ND-H	−2.530500204	0.001151839
*Rothia dentocariosa*	ND-SP vs. ND-H	−5.31488562	9.51749E-12
*Leptotrichia hongkongensis*	ND-SP vs. ND-H	−5.058772642	5.50121E-10
*Porphyromonas pasteri*	ND-SP vs. ND-H	−6.57415721	7.31436E-14
*Rothia aeria*	ND-SP vs. ND-H	−4.702220479	1.04814E-10
*Bifidobacterium longum*	ND-SP vs. ND-H	5.063088721	8.38497E-08
*Actinomyces oris*	ND-SP vs. ND-H	−3.379293088	1.72297E-07
*Rothia mucilaginosa*	ND-SP vs. ND-H	−3.005423076	1.93122E-07
*Actinomyces massiliensis*	ND-SP vs. ND-H	−2.17571579	3.06714E-05
*Bacteroides plebeius*	ND-SP vs. ND-H	−1.732380066	0.000294585
*Leptotrichia massiliensis*	ND-SP vs. ND-H	−2.25588348	8.9826E-06
*Faecalibacterium prausnitzii*	ND-SP vs. ND-H	−3.267475525	0.000371775
*Corynebacterium durum*	ND-SP vs. ND-H	−1.833846965	0.002466958
*Fusobacterium periodonticum*	ND-SP vs. ND-H	−2.898134361	2.03813E-05
*Peptoanaerobacter stomatis*	ND-SP vs. ND-H	1.692188023	0.000793819
*Mogibacterium timidum*	ND-SP vs. ND-H	0.946154515	0.007610078
*Bergeyella cardium*	ND-SP vs. ND-H	−1.532633974	0.003435333
*Helicobacter ganmani*	ND-SP vs. ND-H	2.919509218	0.000186798
*Rhodococcus erythropolis*	ND-SP vs. ND-H	−1.222484116	0.027114593
*Capnocytophaga granulosa*	ND-SP vs. ND-H	−1.442185514	0.03660818
*Filifactor alocis*	T2DM-MP vs. T2DM-H	2.95204757	0.000159082
*Cardiobacterium hominis*	T2DM-MP vs. T2DM-H	−1.3074733	0.035495695
*Tannerella forsythia*	T2DM-MP vs. T2DM-H	3.625678789	1.38449E-05
*Fretibacterium fastidiosum*	T2DM-MP vs. T2DM-H	4.216570358	3.1967E-06
*Eubacterium brachy*	T2DM-MP vs. T2DM-H	3.837283237	7.48867E-06
*Capnocytophaga gingivalis*	T2DM-MP vs. T2DM-H	−2.674502208	0.001958334
*Cutibacterium acnes*	T2DM-MP vs. T2DM-H	−2.897332431	0.001058044
*Porphyromonas gingivalis*	T2DM-MP vs. T2DM-H	5.803304019	4.95825E-06
*Capnocytophaga sputigena*	T2DM-MP vs. T2DM-H	−2.808967355	0.012106292
*Parvimonas micra*	T2DM-MP vs. T2DM-H	2.651223031	0.000382452
*Porphyromonas endodontalis*	T2DM-MP vs. T2DM-H	3.031059138	0.014367338
*Sphingomonas leidyi*	T2DM-MP vs. T2DM-H	−4.729663355	1.85583E-06
*Phocaeicola abscessus*	T2DM-MP vs. T2DM-H	3.696874599	5.82696E-06
*Eubacterium infirmum*	T2DM-MP vs. T2DM-H	1.899346295	0.027470344
*Peptostreptococcus stomatis*	T2DM-MP vs. T2DM-H	2.722978125	0.000466133
*Prevotella intermedia*	T2DM-MP vs. T2DM-H	4.31632738	2.71477E-05
*Eubacterium saphenum*	T2DM-MP vs. T2DM-H	2.42978377	0.022756554
*Oribacterium sinus*	T2DM-MP vs. T2DM-H	−1.398352866	0.000491458
*Fusobacterium simiae*	T2DM-MP vs. T2DM-H	2.589755267	0.003488827
*Phyllobacterium myrsinacearum*	T2DM-MP vs. T2DM-H	−3.524008684	0.000205833
*Asaia bogorensis*	T2DM-MP vs. T2DM-H	−2.534595246	3.19269E-05
*Leptotrichia wadei*	T2DM-MP vs. T2DM-H	3.36649914	0.000117876
*Lachnoanaerobaculum saburreum*	T2DM-MP vs. T2DM-H	2.958723975	0.000351391
*Catonella morbi*	T2DM-MP vs. T2DM-H	1.795562371	0.002143208
*Clostridium celatum*	T2DM-MP vs. T2DM-H	−1.402819141	0.004052787
*Prevotella nigrescens*	T2DM-MP vs. T2DM-H	2.440474438	0.005626979
*Solobacterium moorei*	T2DM-MP vs. T2DM-H	1.670298585	0.00900565
*Campylobacter gracilis*	T2DM-MP vs. T2DM-H	2.119820935	0.012031549
*Fusobacterium nucleatum*	T2DM-MP vs. T2DM-H	0.844362158	0.041788712
*Filifactor alocis*	T2DM-SP vs. T2DM-H	3.030822053	0.000150492
*Cardiobacterium hominis*	T2DM-SP vs. T2DM-H	−2.06437939	0.001688332
*Tannerella forsythia*	T2DM-SP vs. T2DM-H	4.162985381	1.90998E-06
*Fretibacterium fastidiosum*	T2DM-SP vs. T2DM-H	4.315967796	3.1254E-06
*Eubacterium brachy*	T2DM-SP vs. T2DM-H	3.592975081	2.99979E-05
*Capnocytophaga gingivalis*	T2DM-SP vs. T2DM-H	−2.819114715	0.001462038
*Cutibacterium acnes*	T2DM-SP vs. T2DM-H	−3.320771223	0.000297723
*Porphyromonas gingivalis*	T2DM-SP vs. T2DM-H	6.155077318	2.60353E-06
*Capnocytophaga sputigena*	T2DM-SP vs. T2DM-H	−2.975871962	0.00950511
*Parvimonas micra*	T2DM-SP vs. T2DM-H	2.539541329	0.000789131
*Porphyromonas endodontalis*	T2DM-SP vs. T2DM-H	3.117017535	0.013834649
*Sphingomonas leidyi*	T2DM-SP vs. T2DM-H	−4.732161885	2.73349E-06
*Phocaeicola abscessus*	T2DM-SP vs. T2DM-H	3.049262261	0.000142986
*Eubacterium infirmum*	T2DM-SP vs. T2DM-H	1.745625756	0.04626106
*Peptostreptococcus stomatis*	T2DM-SP vs. T2DM-H	2.400261903	0.002131451
*Prevotella intermedia*	T2DM-SP vs. T2DM-H	2.530486321	0.009923158
*Eubacterium saphenum*	T2DM-SP vs. T2DM-H	2.719955848	0.013164662
*Oribacterium sinus*	T2DM-SP vs. T2DM-H	−0.889385648	0.023500566
*Fusobacterium simiae*	T2DM-SP vs. T2DM-H	3.059502786	0.000889993
*Phyllobacterium myrsinacearum*	T2DM-SP vs. T2DM-H	−3.598707857	0.000208535
*Asaia bogorensis*	T2DM-SP vs. T2DM-H	−2.608471024	2.8878E-05
*Leptotrichia wadei*	T2DM-SP vs. T2DM-H	4.010989868	1.25877E-05
*Lachnoanaerobaculum saburreum*	T2DM-SP vs. T2DM-H	2.572991078	0.001926568
*Catonella morbi*	T2DM-SP vs. T2DM-H	1.683781268	0.004515051
*Clostridium celatum*	T2DM-SP vs. T2DM-H	−1.50087272	0.002746956
*Prevotella nigrescens*	T2DM-SP vs. T2DM-H	2.407496861	0.007364875
*Solobacterium moorei*	T2DM-SP vs. T2DM-H	1.463736888	0.023662165
*Campylobacter gracilis*	T2DM-SP vs. T2DM-H	1.933553973	0.023982774
*Fusobacterium nucleatum*	T2DM-SP vs. T2DM-H	0.907165787	0.03284018
Feature	Value	Coef	Pval
*Species significantly differing between the moderate/severe periodontitis groups and the healthy group in the Sal niche*			
*Tannerella forsythia*	*ND-MP vs. ND-H*	*3.446326195*	*1.03925E-07*
*Phocaeicola abscessus*	*ND-MP vs. ND-H*	*2.821445239*	*4.80517E-07*
*Faecalibacterium prausnitzii*	*ND-MP vs. ND-H*	*4.051810651*	*9.42726E-06*
*Peptostreptococcus stomatis*	*ND-MP vs. ND-H*	*1.982563751*	*3.52905E-05*
*Fusobacterium simiae*	*ND-MP vs. ND-H*	*2.396375714*	*0.001130763*
*Lachnoclostridium edouardi*	*ND-MP vs. ND-H*	*1.352129101*	*0.001178649*
*Bacteroides uniformis*	*ND-MP vs. ND-H*	*2.233310712*	*0.002091988*
*Lachnospira pectinoschiza*	*ND-MP vs. ND-H*	*1.928153983*	*0.002038411*
*Parvimonas micra*	*ND-MP vs. ND-H*	*1.642457254*	*0.002449727*
*Filifactor alocis*	*ND-MP vs. ND-H*	*1.616476383*	*0.003015725*
*Eubacterium brachy*	*ND-MP vs. ND-H*	*1.83403472*	*0.004953843*
*Lachnoanaerobaculum cf.*	*ND-MP vs. ND-H*	*0.750613928*	*0.007666902*
*Campylobacter showae*	*ND-MP vs. ND-H*	*1.592243801*	*0.009677107*
*Campylobacter gracilis*	*ND-MP vs. ND-H*	*1.745120793*	*0.010442921*
*Porphyromonas endodontalis*	*ND-MP vs. ND-H*	*1.605011033*	*0.024032186*
*Eubacterium saphenum*	*ND-MP vs. ND-H*	*1.831520042*	*0.041611002*
*Actinomyces oris*	*ND-MP vs. ND-H*	*−3.9864788*	*9.59242E-05*
*Rothia dentocariosa*	*ND-MP vs. ND-H*	*−2.277513015*	*0.000109207*
*Prevotella nigrescens*	*ND-MP vs. ND-H*	*2.010909846*	*0.000400766*
*Lancefieldella parvula*	*ND-MP vs. ND-H*	*−1.914934501*	*0.000521007*
*Cutibacterium acnes*	*ND-MP vs. ND-H*	*2.468124727*	*0.001468218*
*Rothia mucilaginosa*	*ND-MP vs. ND-H*	*−1.708531775*	*0.001839773*
*Fretibacterium fastidiosum*	*ND-MP vs. ND-H*	*1.694247877*	*0.00247956*
*Prevotella denticola*	*ND-MP vs. ND-H*	*2.395083433*	*0.002901707*
*Prevotella micans*	*ND-MP vs. ND-H*	*1.543883993*	*0.003373195*
*Odoribacter splanchnicus*	*ND-MP vs. ND-H*	*1.630230197*	*0.003526973*
*Prevotella histicola*	*ND-MP vs. ND-H*	*−2.43467363*	*0.008220605*
*Blautia obeum*	*ND-MP vs. ND-H*	*1.246643258*	*0.012500466*
*Pyramidobacter piscolens*	*ND-MP vs. ND-H*	*0.792790968*	*0.044238834*
*Tannerella forsythia*	*ND-SP vs. ND-H*	*3.998127269*	*1.84184E-11*
*Phocaeicola abscessus*	*ND-SP vs. ND-H*	*3.321823401*	*1.0364E-10*
*Faecalibacterium prausnitzii*	*ND-SP vs. ND-H*	*3.629253351*	*5.84058E-06*
*Peptostreptococcus stomatis*	*ND-SP vs. ND-H*	*2.377413529*	*4.49741E-08*
*Fusobacterium simiae*	*ND-SP vs. ND-H*	*2.655997945*	*4.85222E-05*
*Lachnoclostridium edouardi*	*ND-SP vs. ND-H*	*0.978563386*	*0.006662605*
*Bacteroides uniformis*	*ND-SP vs. ND-H*	*1.432996871*	*0.022106486*
*Lachnospira pectinoschiza*	*ND-SP vs. ND-H*	*1.475007845*	*0.006550366*
*Parvimonas micra*	*ND-SP vs. ND-H*	*1.700598834*	*0.000375704*
*Filifactor alocis*	*ND-SP vs. ND-H*	*2.591158184*	*2.36483E-07*
*Eubacterium brachy*	*ND-SP vs. ND-H*	*2.751375572*	*3.4741E-06*
*Lachnoanaerobaculum cf.*	*ND-SP vs. ND-H*	*1.04681139*	*3.51223E-05*
*Campylobacter showae*	*ND-SP vs. ND-H*	*2.313916161*	*2.98601E-05*
*Campylobacter gracilis*	*ND-SP vs. ND-H*	*1.66156958*	*0.005387819*
*Porphyromonas endodontalis*	*ND-SP vs. ND-H*	*1.548703301*	*0.012919207*
*Eubacterium saphenum*	*ND-SP vs. ND-H*	*3.099213564*	*0.000127739*
*Actinomyces oris*	*ND-SP vs. ND-H*	*−3.106574293*	*0.000447292*
*Rothia dentocariosa*	*ND-SP vs. ND-H*	*−1.434462451*	*0.004417384*
*Prevotella nigrescens*	*ND-SP vs. ND-H*	*1.425871321*	*0.00363503*
*Lancefieldella parvula*	*ND-SP vs. ND-H*	*−1.641040352*	*0.000651055*
*Cutibacterium acnes*	*ND-SP vs. ND-H*	*2.30443048*	*0.000704043*
*Rothia mucilaginosa*	*ND-SP vs. ND-H*	*−1.084405423*	*0.021836556*
*Fretibacterium fastidiosum*	*ND-SP vs. ND-H*	*2.542620253*	*6.68359E-07*
*Prevotella denticola*	*ND-SP vs. ND-H*	*1.64232666*	*0.018237883*
*Prevotella micans*	*ND-SP vs. ND-H*	*1.535378752*	*0.000913665*
*Odoribacter splanchnicus*	*ND-SP vs. ND-H*	*1.165636475*	*0.016049902*
*Prevotella histicola*	*ND-SP vs. ND-H*	*−2.925650755*	*0.000347715*
*Blautia obeum*	*ND-SP vs. ND-H*	*1.300165513*	*0.003059146*
*Pyramidobacter piscolens*	*ND-SP vs. ND-H*	*0.831324474*	*0.016211169*
*Tannerella forsythia*	*T2DM-MP vs. T2DM-H*	*1.556665207*	*0.010644688*
*Phocaeicola abscessus*	*T2DM-MP vs. T2DM-H*	*1.958586021*	*0.011842736*
*Faecalibacterium prausnitzii*	*T2DM-MP vs. T2DM-H*	*3.571903223*	*0.001787747*
*Peptostreptococcus stomatis*	*T2DM-MP vs. T2DM-H*	*2.474711711*	*0.002414986*
*Fusobacterium simiae*	*T2DM-MP vs. T2DM-H*	*2.620650972*	*0.001312562*
*Lachnoclostridium edouardi*	*T2DM-MP vs. T2DM-H*	*1.933006029*	*0.007227312*
*Bacteroides uniformis*	*T2DM-MP vs. T2DM-H*	*1.916707039*	*0.026256452*
*Lachnospira pectinoschiza*	*T2DM-MP vs. T2DM-H*	*2.66528588*	*0.010202793*
*Parvimonas micra*	*T2DM-MP vs. T2DM-H*	*2.231691943*	*0.005291807*
*Filifactor alocis*	*T2DM-MP vs. T2DM-H*	*1.512574829*	*0.010234203*
*Eubacterium brachy*	*T2DM-MP vs. T2DM-H*	*2.001141022*	*0.008271434*
*Lachnoanaerobaculum cf.*	*T2DM-MP vs. T2DM-H*	*1.321054036*	*0.009671342*
*Campylobacter showae*	*T2DM-MP vs. T2DM-H*	*1.338281554*	*0.046054438*
*Campylobacter gracilis*	*T2DM-MP vs. T2DM-H*	*2.123260996*	*0.017878525*
*Porphyromonas endodontalis*	*T2DM-MP vs. T2DM-H*	*1.935600716*	*0.005349517*
*Eubacterium saphenum*	*T2DM-MP vs. T2DM-H*	*2.905713552*	*0.002441905*
*Leptotrichia wadei*	*T2DM-MP vs. T2DM-H*	*3.003737885*	*0.00024671*
*Capnocytophaga granulosa*	*T2DM-MP vs. T2DM-H*	*1.67584246*	*0.010893133*
*Leptotrichia hofstadii*	*T2DM-MP vs. T2DM-H*	*1.520016592*	*0.011733401*
*Fusobacterium periodonticum*	*T2DM-MP vs. T2DM-H*	*−1.211213225*	*0.020906234*
*Tannerella forsythia*	*T2DM-SP vs. T2DM-H*	*1.899805021*	*0.00262135*
*Phocaeicola abscessus*	*T2DM-SP vs. T2DM-H*	*1.889760978*	*0.017242497*
*Faecalibacterium prausnitzii*	*T2DM-SP vs. T2DM-H*	*3.6328921*	*0.001890937*
*Peptostreptococcus stomatis*	*T2DM-SP vs. T2DM-H*	*2.302543585*	*0.005442828*
*Fusobacterium simiae*	*T2DM-SP vs. T2DM-H*	*2.936833019*	*0.000487503*
*Lachnoclostridium edouardi*	*T2DM-SP vs. T2DM-H*	*1.939932569*	*0.008328132*
*Bacteroides uniformis*	*T2DM-SP vs. T2DM-H*	*1.730637548*	*0.048648147*
*Lachnospira pectinoschiza*	*T2DM-SP vs. T2DM-H*	*2.778204566*	*0.008938343*
*Parvimonas micra*	*T2DM-SP vs. T2DM-H*	*2.511724302*	*0.002324962*
*Filifactor alocis*	*T2DM-SP vs. T2DM-H*	*1.780607536*	*0.003432548*
*Eubacterium brachy*	*T2DM-SP vs. T2DM-H*	*2.019781262*	*0.009116585*
*Lachnoanaerobaculum cf.*	*T2DM-SP vs. T2DM-H*	*1.522849981*	*0.003834465*
*Campylobacter showae*	*T2DM-SP vs. T2DM-H*	*2.081663027*	*0.003055393*
*Campylobacter gracilis*	*T2DM-SP vs. T2DM-H*	*2.785210797*	*0.00281719*
*Porphyromonas endodontalis*	*T2DM-SP vs. T2DM-H*	*2.044514602*	*0.004118293*
*Eubacterium saphenum*	*T2DM-SP vs. T2DM-H*	*2.858863349*	*0.003458543*
*Leptotrichia wadei*	*T2DM-SP vs. T2DM-H*	*3.926768721*	*6.18783E-06*
*Capnocytophaga granulosa*	*T2DM-SP vs. T2DM-H*	*2.503855404*	*0.000318142*
*Leptotrichia hofstadii*	*T2DM-SP vs. T2DM-H*	*2.149310073*	*0.000687459*
*Fusobacterium periodonticum*	*T2DM-SP vs. T2DM-H*	*−1.48272824*	*0.006244559*

### Appendix Table 3

**Table A3.** Significant mediation effects observed in ND or T2DM subjects.

x	Factor	y
*Significant mediation effects observed in ND subjects*		
*Filifactor alocis*	BI	Disease State
*Filifactor alocis*	PLI	Disease State
*Filifactor alocis*	GI	Disease State
*Filifactor alocis*	PD	Disease State
*Filifactor alocis*	CAL	Disease State
*Phocaeicola abscessus*	BI	Disease State
*Phocaeicola abscessus*	PLI	Disease State
*Phocaeicola abscessus*	GI	Disease State
*Phocaeicola abscessus*	PD	Disease State
*Phocaeicola abscessus*	CAL	Disease State
*Porphyromonas gingivalis*	BI	Disease State
*Porphyromonas gingivalis*	PLI	Disease State
*Porphyromonas gingivalis*	GI	Disease State
*Porphyromonas gingivalis*	PD	Disease State
*Porphyromonas gingivalis*	CAL	Disease State
*Tannerella forsythia*	BI	Disease State
*Tannerella forsythia*	PLI	Disease State
*Tannerella forsythia*	GI	Disease State
*Tannerella forsythia*	PD	Disease State
*Tannerella forsythia*	CAL	Disease State
*Parvimonas micra*	BI	Disease State
*Parvimonas micra*	PLI	Disease State
*Parvimonas micra*	GI	Disease State
*Parvimonas micra*	PD	Disease State
*Parvimonas micra*	CAL	Disease State
*Alloprevotella tannerae*	BI	Disease State
*Alloprevotella tannerae*	GI	Disease State
*Eubacterium brachy*	BI	Disease State
*Eubacterium brachy*	PLI	Disease State
*Eubacterium brachy*	GI	Disease State
*Eubacterium brachy*	PD	Disease State
*Eubacterium brachy*	CAL	Disease State
*Peptoanaerobacter stomatis*	BI	Disease State
*Peptoanaerobacter stomatis*	PLI	Disease State
*Peptoanaerobacter stomatis*	GI	Disease State
*Peptoanaerobacter stomatis*	PD	Disease State
*Peptoanaerobacter stomatis*	CAL	Disease State
*Pseudoramibacter alactolyticus*	BI	Disease State
*Pseudoramibacter alactolyticus*	PLI	Disease State
*Pseudoramibacter alactolyticus*	GI	Disease State
*Pseudoramibacter alactolyticus*	PD	Disease State
*Pseudoramibacter alactolyticus*	CAL	Disease State
*Eubacterium infirmum*	GI	Disease State
*Porphyromonas pasteri*	PLI	Disease State
*Porphyromonas pasteri*	PD	Disease State
*Porphyromonas pasteri*	CAL	Disease State
*Corynebacterium durum*	BI	Disease State
*Corynebacterium durum*	PLI	Disease State
*Corynebacterium durum*	GI	Disease State
*Corynebacterium durum*	PD	Disease State
*Corynebacterium durum*	CAL	Disease State
*Rothia dentocariosa*	BI	Disease State
*Rothia dentocariosa*	PLI	Disease State
*Rothia dentocariosa*	GI	Disease State
*Rothia dentocariosa*	PD	Disease State
*Rothia dentocariosa*	CAL	Disease State
*Actinomyces oris*	BI	Disease State
*Actinomyces oris*	PLI	Disease State
*Actinomyces oris*	PD	Disease State
*Actinomyces oris*	CAL	Disease State
*Actinomyces naeslundii*	BI	Disease State
*Actinomyces naeslundii*	PLI	Disease State
*Actinomyces naeslundii*	GI	Disease State
*Actinomyces naeslundii*	PD	Disease State
*Actinomyces naeslundii*	CAL	Disease State
*Anaerostipes hadrus*	PLI	Disease State
*Anaerostipes hadrus*	GI	Disease State
*Anaerostipes hadrus*	CAL	Disease State
*Blautia obeum*	PLI	Disease State
*Bacteroides ovatus*	PLI	Disease State
*Bacteroides ovatus*	CAL	Disease State
*Leptotrichia hofstadii*	BI	Disease State
*Leptotrichia hofstadii*	PLI	Disease State
*Leptotrichia hofstadii*	GI	Disease State
*Leptotrichia hofstadii*	PD	Disease State
*Leptotrichia hofstadii*	CAL	Disease State
*Actinomyces massiliensis*	BI	Disease State
*Actinomyces massiliensis*	PLI	Disease State
*Actinomyces massiliensis*	GI	Disease State
*Actinomyces massiliensis*	PD	Disease State
*Actinomyces massiliensis*	CAL	Disease State
*Brevundimonas vesicularis*	BI	Disease State
*Brevundimonas vesicularis*	PLI	Disease State
*Brevundimonas vesicularis*	GI	Disease State
*Brevundimonas vesicularis*	PD	Disease State
*Brevundimonas vesicularis*	CAL	Disease State
*Streptacidiphilus jiangxiensis*	BI	Disease State
*Streptacidiphilus jiangxiensis*	PLI	Disease State
*Streptacidiphilus jiangxiensis*	GI	Disease State
*Streptacidiphilus jiangxiensis*	PD	Disease State
*Streptacidiphilus jiangxiensis*	CAL	Disease State
*Streptacidiphilus rugosus*	BI	Disease State
*Streptacidiphilus rugosus*	PLI	Disease State
*Streptacidiphilus rugosus*	GI	Disease State
*Streptacidiphilus rugosus*	PD	Disease State
*Streptacidiphilus rugosus*	CAL	Disease State
*Catenulispora acidiphila*	BI	Disease State
*Catenulispora acidiphila*	PLI	Disease State
*Catenulispora acidiphila*	GI	Disease State
*Catenulispora acidiphila*	PD	Disease State
*Catenulispora acidiphila*	CAL	Disease State
*Streptacidiphilus pinicola*	BI	Disease State
*Streptacidiphilus pinicola*	PLI	Disease State
*Streptacidiphilus pinicola*	GI	Disease State
*Streptacidiphilus pinicola*	PD	Disease State
*Streptacidiphilus pinicola*	CAL	Disease State
*Actinomyces gerencseriae*	BI	Disease State
*Actinomyces gerencseriae*	GI	Disease State
*Actinomyces gerencseriae*	PD	Disease State
*Actinomyces gerencseriae*	CAL	Disease State
*Helicobacter ganmani*	PLI	Disease State
*Helicobacter ganmani*	CAL	Disease State
*Helicobacter ganmani*	HbA1C	Disease State
*Rhodococcus erythropolis*	BI	Disease State
*Rhodococcus erythropolis*	PLI	Disease State
*Rhodococcus erythropolis*	PD	Disease State
*Rhodococcus erythropolis*	CAL	Disease State
*Cardiobacterium hominis*	BI	Disease State
*Cardiobacterium hominis*	PLI	Disease State
*Cardiobacterium hominis*	GI	Disease State
*Cardiobacterium hominis*	PD	Disease State
*Cardiobacterium hominis*	CAL	Disease State
*Phyllobacterium myrsinacearum*	PLI	Disease State
*Phyllobacterium myrsinacearum*	GI	Disease State
*Phyllobacterium myrsinacearum*	PD	Disease State
*Phyllobacterium myrsinacearum*	CAL	Disease State
*Sphingomonas leidyi*	BI	Disease State
*Sphingomonas leidyi*	PLI	Disease State
*Sphingomonas leidyi*	CAL	Disease State
*Mediterraneibacter faecis*	BI	Disease State
*Mediterraneibacter faecis*	PLI	Disease State
*Mediterraneibacter faecis*	PD	Disease State
*Mediterraneibacter faecis*	CAL	Disease State
*Bifidobacterium longum*	PLI	Disease State
*Bifidobacterium longum*	PD	Disease State
*Bifidobacterium longum*	CAL	Disease State
*Bifidobacterium catenulatum*	BI	Disease State
*Bifidobacterium catenulatum*	PLI	Disease State
*Bifidobacterium catenulatum*	GI	Disease State
*Bifidobacterium catenulatum*	CAL	Disease State
*Porphyromonas gulae*	BI	Disease State
*Porphyromonas gulae*	GI	Disease State
*Porphyromonas gulae*	PD	Disease State
*Porphyromonas gulae*	CAL	Disease State
*Campylobacter showae*	GI	Disease State
*Campylobacter showae*	PD	Disease State
*Campylobacter showae*	CAL	Disease State
*Prevotella intermedia*	BI	Disease State
*Prevotella intermedia*	CAL	Disease State
*Fusobacterium nucleatum*	BI	Disease State
*Fusobacterium nucleatum*	PLI	Disease State
*Fusobacterium nucleatum*	GI	Disease State
*Fusobacterium nucleatum*	PD	Disease State
*Fusobacterium nucleatum*	CAL	Disease State
*Scardovia wiggsiae*	BI	Disease State
*Scardovia wiggsiae*	GI	Disease State
*Fusobacterium simiae*	BI	Disease State
*Fusobacterium simiae*	GI	Disease State
*Fusobacterium simiae*	PD	Disease State
*Fusobacterium simiae*	CAL	Disease State
*Pyramidobacter piscolens*	BI	Disease State
*Pyramidobacter piscolens*	PLI	Disease State
*Pyramidobacter piscolens*	GI	Disease State
*Olsenella uli*	PLI	Disease State
*Porphyromonas endodontalis*	BI	Disease State
*Porphyromonas endodontalis*	PLI	Disease State
*Porphyromonas endodontalis*	GI	Disease State
*Porphyromonas endodontalis*	PD	Disease State
*Porphyromonas endodontalis*	CAL	Disease State
*Catonella morbi*	BI	Disease State
*Catonella morbi*	PLI	Disease State
*Catonella morbi*	GI	Disease State
*Catonella morbi*	PD	Disease State
*Catonella morbi*	CAL	Disease State
x	Factor	y
*Significant mediation effects observed in T2DM subjects*		
*Parvimonas micra*	BI	Disease State
*Parvimonas micra*	CAL	Disease State
*Prevotella nigrescens*	BI	Disease State
*Prevotella nigrescens*	GI	Disease State
*Prevotella nigrescens*	PD	Disease State
*Prevotella nigrescens*	CAL	Disease State
*Prevotella veroralis*	BI	Disease State
*Prevotella veroralis*	GI	Disease State
*Pseudoramibacter alactolyticus*	BI	Disease State
*Pseudoramibacter alactolyticus*	PD	Disease State
*Pseudoramibacter alactolyticus*	CAL	Disease State
*Eubacterium infirmum*	BI	Disease State
*Eubacterium infirmum*	PLI	Disease State
*Eubacterium infirmum*	GI	Disease State
*Eubacterium infirmum*	CAL	Disease State
*Stomatobaculum longum*	BI	Disease State
*Stomatobaculum longum*	GI	Disease State
*Stomatobaculum longum*	PD	Disease State
*Stomatobaculum longum*	CAL	Disease State
*Solobacterium moorei*	BI	Disease State
*Solobacterium moorei*	PD	Disease State
*Porphyromonas pasteri*	BI	Disease State
*Porphyromonas pasteri*	PLI	Disease State
*Porphyromonas pasteri*	GI	Disease State
*Porphyromonas pasteri*	PD	Disease State
*Porphyromonas pasteri*	CAL	Disease State
*Fusobacterium nucleatum*	GI	Disease State
*Fusobacterium nucleatum*	CAL	Disease State
*Fusobacterium simiae*	PD	Disease State
*Fusobacterium simiae*	CAL	Disease State
*Pyramidobacter piscolens*	BI	Disease State
*Pyramidobacter piscolens*	PLI	Disease State
*Pyramidobacter piscolens*	PD	Disease State
*Capnocytophaga sputigena*	BI	Disease State
*Capnocytophaga sputigena*	PLI	Disease State
*Capnocytophaga sputigena*	CAL	Disease State
*Fusobacterium hwasookii*	BI	Disease State
*Fusobacterium hwasookii*	GI	Disease State
*Fusobacterium hwasookii*	PD	Disease State
*Fusobacterium hwasookii*	CAL	Disease State

### Appendix Table 4

**Table A4.** Significant mediation effects observed in ND or T2DM subjects.

Feature	Value	Coef	Pval
*Species significantly differing between Post-Tx groups and Pre-Tx group in the Pla niche*			
*Phocaeicola abscessus*	Post-Tx vs. Pre-Tx (Periodontitis)	*−4.859268625*	2.38938E-09
*Porphyromonas endodontalis*	Post-Tx vs. Pre-Tx (Periodontitis)	*−5.918928073*	2.49E-09
*Catonella morbi*	Post-Tx vs. Pre-Tx (Periodontitis)	*−2.776238473*	1.09704E-07
*Phyllobacterium myrsinacearum*	Post-Tx vs. Pre-Tx (Periodontitis)	*3.610690517*	1.59684E-07
*Peptoanaerobacter stomatis*	Post-Tx vs. Pre-Tx (Periodontitis)	*−3.441621764*	2.15178E-07
*Eubacterium saphenum*	Post-Tx vs. Pre-Tx (Periodontitis)	*−4.957669869*	4.01995E-07
*Parvimonas micra*	Post-Tx vs. Pre-Tx (Periodontitis)	*−2.732561254*	4.87268E-07
*Sphingomonas leidyi*	Post-Tx vs. Pre-Tx (Periodontitis)	*2.844053681*	5.35578E-07
*Brevundimonas vesicularis*	Post-Tx vs. Pre-Tx (Periodontitis)	*2.744935968*	1.06483E-06
*Filifactor alocis*	Post-Tx vs. Pre-Tx (Periodontitis)	*−4.082907438*	1.00344E-06
*Tannerella forsythia*	Post-Tx vs. Pre-Tx (Periodontitis)	*−4.693810366*	1.75156E-06
*Eubacterium brachy*	Post-Tx vs. Pre-Tx (Periodontitis)	*−4.098228386*	2.14038E-06
*Fretibacterium fastidiosum*	Post-Tx vs. Pre-Tx (Periodontitis)	*−4.54763073*	2.73043E-06
*Porphyromonas gingivalis*	Post-Tx vs. Pre-Tx (Periodontitis)	*−4.54953324*	1.0732E-05
*Peptostreptococcus stomatis*	Post-Tx vs. Pre-Tx (Periodontitis)	*−2.504657907*	3.5969E-05
*Fusobacterium nucleatum*	Post-Tx vs. Pre-Tx (Periodontitis)	*−2.506301923*	5.63585E-05
*Campylobacter showae*	Post-Tx vs. Pre-Tx (Periodontitis)	*−3.148512405*	9.28111E-05
*Prevotella veroralis*	Post-Tx vs. Pre-Tx (Periodontitis)	*−1.378196125*	0.000112068
*Pyramidobacter piscolens*	Post-Tx vs. Pre-Tx (Periodontitis)	*−2.210981068*	0.000191291
*Eubacterium yurii*	Post-Tx vs. Pre-Tx (Periodontitis)	*−2.097209869*	0.000215218
*Schaalia odontolytica*	Post-Tx vs. Pre-Tx (Periodontitis)	*3.240006343*	0.000291937
*Leptotrichia wadei*	Post-Tx vs. Pre-Tx (Periodontitis)	*−2.903964964*	0.000391782
*Mogibacterium timidum*	Post-Tx vs. Pre-Tx (Periodontitis)	*−1.328618791*	0.000554844
*Prevotella intermedia*	Post-Tx vs. Pre-Tx (Periodontitis)	*−4.553440871*	0.001118181
*Campylobacter gracilis*	Post-Tx vs. Pre-Tx (Periodontitis)	*−2.539475486*	0.001277136
*Campylobacter concisus*	Post-Tx vs. Pre-Tx (Periodontitis)	*−2.011134904*	0.00417351
*Prevotella denticola*	Post-Tx vs. Pre-Tx (Periodontitis)	*−2.348455493*	0.004200466
*Fusobacterium simiae*	Post-Tx vs. Pre-Tx (Periodontitis)	*−2.486721589*	0.004820949
*Scardovia wiggsiae*	Post-Tx vs. Pre-Tx (Periodontitis)	*−1.23672497*	0.005122535
*Alloprevotella tannerae*	Post-Tx vs. Pre-Tx (Periodontitis)	*−1.728983409*	0.005683122
*Bifidobacterium longum*	Post-Tx vs. Pre-Tx (Periodontitis)	*1.589164904*	0.005543641
*Prevotella nigrescens*	Post-Tx vs. Pre-Tx (Periodontitis)	*−2.247484393*	0.005409419
*Fusobacterium hwasookii*	Post-Tx vs. Pre-Tx (Periodontitis)	*−1.815143164*	0.009246109
*Stomatobaculum longum*	Post-Tx vs. Pre-Tx (Periodontitis)	*−1.35392478*	0.009339355
*Lancefieldella rimae*	Post-Tx vs. Pre-Tx (Periodontitis)	*2.282867072*	0.010692624
*Olsenella uli*	Post-Tx vs. Pre-Tx (Periodontitis)	*−1.664966126*	0.011056969
*Bifidobacterium catenulatum*	Post-Tx vs. Pre-Tx (Periodontitis)	*−0.88288384*	0.011528917
*Bulleidia extructa*	Post-Tx vs. Pre-Tx (Periodontitis)	*−0.823699533*	0.012768364
*Lachnoanaerobaculum saburreum*	Post-Tx vs. Pre-Tx (Periodontitis)	*−1.381250581*	0.012823803
*Eubacterium infirmum*	Post-Tx vs. Pre-Tx (Periodontitis)	*−1.021780658*	0.013830747
*Johnsonella ignava*	Post-Tx vs. Pre-Tx (Periodontitis)	*−0.997501769*	0.014361432
*Fusobacterium periodonticum*	Post-Tx vs. Pre-Tx (Periodontitis)	*−1.463874919*	0.019922744
*Porphyromonas gulae*	Post-Tx vs. Pre-Tx (Periodontitis)	*−0.9545037*	0.021791898
*Leptotrichia buccalis*	Post-Tx vs. Pre-Tx (Periodontitis)	*−1.273382672*	0.027962794
*Prevotella micans*	Post-Tx vs. Pre-Tx (Periodontitis)	*−1.522967929*	0.029795908
*Olsenella phocaeensis*	Post-Tx vs. Pre-Tx (Periodontitis)	*−0.67811288*	0.034111892
*Shuttleworthia satelles*	Post-Tx vs. Pre-Tx (Periodontitis)	*−0.311671966*	0.039940362
*Capnocytophaga gingivalis*	Post-Tx vs. Pre-Tx (Periodontitis)	*1.51795676*	0.045018738
*Pseudoramibacter alactolyticus*	Post-Tx vs. Pre-Tx (Periodontitis)	*−1.427158957*	0.048322652
*Bergeyella cardium*	Post-Tx vs. Pre-Tx (T2DM)	*2.691803988*	2.99711E-06
*Peptoanaerobacter stomatis*	Post-Tx vs. Pre-Tx (T2DM)	*−2.017373038*	4.03188E-06
*Stomatobaculum longum*	Post-Tx vs. Pre-Tx (T2DM)	*−1.772025019*	6.24368E-06
*Faecalibacterium prausnitzii*	Post-Tx vs. Pre-Tx (T2DM)	*−2.396092912*	1.68497E-05
*Slackia exigua*	Post-Tx vs. Pre-Tx (T2DM)	*−1.366325169*	2.44608E-05
*Bifidobacterium catenulatum*	Post-Tx vs. Pre-Tx (T2DM)	*−1.783864347*	0.000177533
*Porphyromonas pasteri*	Post-Tx vs. Pre-Tx (T2DM)	*1.500452073*	0.000192049
*Solobacterium moorei*	Post-Tx vs. Pre-Tx (T2DM)	*−0.952156361*	0.00019358
*Olsenella uli*	Post-Tx vs. Pre-Tx (T2DM)	*−1.591825039*	0.000371782
*Fusobacterium hwasookii*	Post-Tx vs. Pre-Tx (T2DM)	*1.272655328*	0.000453859
*Scardovia wiggsiae*	Post-Tx vs. Pre-Tx (T2DM)	*−2.432713069*	0.00079576
*Blautia obeum*	Post-Tx vs. Pre-Tx (T2DM)	*−1.630403367*	0.001264052
*Prevotella veroralis*	Post-Tx vs. Pre-Tx (T2DM)	*−1.538266705*	0.00228416
*Parvimonas micra*	Post-Tx vs. Pre-Tx (T2DM)	*0.825590942*	0.003307442
*Lancefieldella parvula*	Post-Tx vs. Pre-Tx (T2DM)	*−0.979398261*	0.006167377
*Prevotella denticola*	Post-Tx vs. Pre-Tx (T2DM)	*−1.453918554*	0.007055638
*Fusobacterium nucleatum*	Post-Tx vs. Pre-Tx (T2DM)	*0.463385838*	0.008724217
*Johnsonella ignava*	Post-Tx vs. Pre-Tx (T2DM)	*1.207988844*	0.00810219
*Lachnoanaerobaculum cf.*	Post-Tx vs. Pre-Tx (T2DM)	*0.748148896*	0.008779039
*Eubacterium yurii*	Post-Tx vs. Pre-Tx (T2DM)	*1.055035575*	0.019167174
*Fusicatenibacter saccharivorans*	Post-Tx vs. Pre-Tx (T2DM)	*−0.806053583*	0.016096569
*Prevotella fusca*	Post-Tx vs. Pre-Tx (T2DM)	*−1.065488461*	0.026655478
*Leptotrichia massiliensis*	Post-Tx vs. Pre-Tx (T2DM)	*0.840306733*	0.039241718
*Leptotrichia wadei*	Post-Tx vs. Pre-Tx (T2DM)	*−0.587838484*	0.041076427
*Prevotella histicola*	Post-Tx vs. Pre-Tx (T2DM)	*−0.600850078*	0.045396923
Feature	Value	Coef	Pval
*Species significantly differing between Post-Tx groups and Pre-Tx group in the GCF niche*			
*Faecalibacterium prausnitzii*	Post-Tx vs. Pre-Tx (Periodontitis)	*5.962859802*	3.49288E-08
*Blautia obeum*	Post-Tx vs. Pre-Tx (Periodontitis)	*3.924180183*	2.4948E-05
*Capnocytophaga gingivalis*	Post-Tx vs. Pre-Tx (Periodontitis)	*3.15860113*	0.000133541
*Cutibacterium acnes*	Post-Tx vs. Pre-Tx (Periodontitis)	*2.546109117*	0.000141317
*Rothia mucilaginosa*	Post-Tx vs. Pre-Tx (Periodontitis)	*3.763006724*	0.000150378
*Phocaeicola abscessus*	Post-Tx vs. Pre-Tx (Periodontitis)	*−4.093188064*	0.000306009
*Porphyromonas gingivalis*	Post-Tx vs. Pre-Tx (Periodontitis)	*−6.840498132*	0.000622571
*Anaerostipes hadrus*	Post-Tx vs. Pre-Tx (Periodontitis)	*3.381527346*	0.00084493
*Capnocytophaga sputigena*	Post-Tx vs. Pre-Tx (Periodontitis)	*2.614404176*	0.000887349
*Rothia aeria*	Post-Tx vs. Pre-Tx (Periodontitis)	*3.927941602*	0.00101858
*Bacteroides vulgatus*	Post-Tx vs. Pre-Tx (Periodontitis)	*2.274966913*	0.001205429
*Filifactor alocis*	Post-Tx vs. Pre-Tx (Periodontitis)	*−5.167076725*	0.001375171
*Oribacterium sinus*	Post-Tx vs. Pre-Tx (Periodontitis)	*2.065157665*	0.001369641
*Fretibacterium fastidiosum*	Post-Tx vs. Pre-Tx (Periodontitis)	*−4.142200228*	0.002395547
*Thermus thermophilus*	Post-Tx vs. Pre-Tx (Periodontitis)	*−0.99972439*	0.002333576
*Mediterraneibacter faecis*	Post-Tx vs. Pre-Tx (Periodontitis)	*2.197912459*	0.00255768
*Eubacterium sulci*	Post-Tx vs. Pre-Tx (Periodontitis)	*1.60015379*	0.006456769
*Bifidobacterium catenulatum*	Post-Tx vs. Pre-Tx (Periodontitis)	*1.998738228*	0.006717361
*Sphingomonas leidyi*	Post-Tx vs. Pre-Tx (Periodontitis)	*3.469233734*	0.006144125
*Erysipelotrichaceae UCG 003 bacterium*	Post-Tx vs. Pre-Tx (Periodontitis)	*1.767622516*	0.008422342
*Lancefieldella parvula*	Post-Tx vs. Pre-Tx (Periodontitis)	*2.165301654*	0.008405554
*Prevotella veroralis*	Post-Tx vs. Pre-Tx (Periodontitis)	*2.974918974*	0.007323943
*Schaalia odontolytica*	Post-Tx vs. Pre-Tx (Periodontitis)	*2.185406794*	0.008378035
*Bacteroides plebeius*	Post-Tx vs. Pre-Tx (Periodontitis)	*1.928003754*	0.009646775
*Cardiobacterium hominis*	Post-Tx vs. Pre-Tx (Periodontitis)	*2.125810548*	0.009873554
*Prevotella intermedia*	Post-Tx vs. Pre-Tx (Periodontitis)	*−4.111065736*	0.010138732
*Romboutsia ilealis*	Post-Tx vs. Pre-Tx (Periodontitis)	*1.749536565*	0.010250719
*Tannerella forsythia*	Post-Tx vs. Pre-Tx (Periodontitis)	*−3.392623513*	0.010419807
*Fusicatenibacter saccharivorans*	Post-Tx vs. Pre-Tx (Periodontitis)	*1.483602251*	0.012930589
*Lachnospira pectinoschiza*	Post-Tx vs. Pre-Tx (Periodontitis)	*1.045895874*	0.013329833
*Campylobacter showae*	Post-Tx vs. Pre-Tx (Periodontitis)	*−2.872153311*	0.014258802
*Prevotella melaninogenica*	Post-Tx vs. Pre-Tx (Periodontitis)	*2.611189152*	0.019053809
*Eubacterium brachy*	Post-Tx vs. Pre-Tx (Periodontitis)	*−2.53260154*	0.021935324
*Porphyromonas pasteri*	Post-Tx vs. Pre-Tx (Periodontitis)	*2.354382491*	0.025707655
*Fusobacterium periodonticum*	Post-Tx vs. Pre-Tx (Periodontitis)	*2.27642489*	0.028521464
*Akkermansia muciniphila*	Post-Tx vs. Pre-Tx (Periodontitis)	*1.242637815*	0.029289972
*Mogibacterium timidum*	Post-Tx vs. Pre-Tx (Periodontitis)	*−1.17903338*	0.029980649
*Peptoanaerobacter stomatis*	Post-Tx vs. Pre-Tx (Periodontitis)	*−1.687690863*	0.030295038
*Alloprevotella tannerae*	Post-Tx vs. Pre-Tx (Periodontitis)	*−2.115513286*	0.037520174
*Campylobacter gracilis*	Post-Tx vs. Pre-Tx (Periodontitis)	*−2.463713554*	0.043208734
*Phyllobacterium myrsinacearum*	Post-Tx vs. Pre-Tx (Periodontitis)	*2.479726132*	0.046023137
*Actinomyces massiliensis*	Post-Tx vs. Pre-Tx (T2DM)	*1.58513995*	4.67172E-07
*Rothia aeria*	Post-Tx vs. Pre-Tx (T2DM)	*2.31013195*	1.06399E-06
*Fusobacterium hwasookii*	Post-Tx vs. Pre-Tx (T2DM)	*1.573741002*	1.56672E-05
*Pyramidobacter piscolens*	Post-Tx vs. Pre-Tx (T2DM)	*−2.557938432*	3.11585E-05
*Prevotella jejuni*	Post-Tx vs. Pre-Tx (T2DM)	*1.809206688*	5.36207E-05
*Fretibacterium fastidiosum*	Post-Tx vs. Pre-Tx (T2DM)	*−1.398983334*	0.000203666
*Prevotella micans*	Post-Tx vs. Pre-Tx (T2DM)	*1.640373638*	0.000548717
*Rothia mucilaginosa*	Post-Tx vs. Pre-Tx (T2DM)	*1.774795485*	0.000524085
*Rothia dentocariosa*	Post-Tx vs. Pre-Tx (T2DM)	*1.919823766*	0.000944418
*Phocaeicola abscessus*	Post-Tx vs. Pre-Tx (T2DM)	*−1.251932161*	0.001139559
*Actinomyces naeslundii*	Post-Tx vs. Pre-Tx (T2DM)	*1.291957913*	0.001333987
*Scardovia wiggsiae*	Post-Tx vs. Pre-Tx (T2DM)	*0.953092568*	0.001432445
*Cardiobacterium hominis*	Post-Tx vs. Pre-Tx (T2DM)	*1.088015625*	0.001974081
*Porphyromonas endodontalis*	Post-Tx vs. Pre-Tx (T2DM)	*−1.618700322*	0.003266894
*Schaalia odontolytica*	Post-Tx vs. Pre-Tx (T2DM)	*1.420743472*	0.003518904
*Pseudoramibacter alactolyticus*	Post-Tx vs. Pre-Tx (T2DM)	*−1.03214153*	0.004772913
*Solobacterium moorei*	Post-Tx vs. Pre-Tx (T2DM)	*0.863067442*	0.004663227
*Tannerella forsythia*	Post-Tx vs. Pre-Tx (T2DM)	*−0.949180849*	0.00473401
*Bacteroides vulgatus*	Post-Tx vs. Pre-Tx (T2DM)	*−1.222539103*	0.012594812
*Capnocytophaga haemolytica*	Post-Tx vs. Pre-Tx (T2DM)	*0.534111197*	0.013887909
*Porphyromonas gingivalis*	Post-Tx vs. Pre-Tx (T2DM)	*−1.186318221*	0.013378534
*Rhodococcus erythropolis*	Post-Tx vs. Pre-Tx (T2DM)	*0.677690593*	0.014135193
*Capnocytophaga gingivalis*	Post-Tx vs. Pre-Tx (T2DM)	*0.913102527*	0.018173615
*Peptostreptococcus stomatis*	Post-Tx vs. Pre-Tx (T2DM)	*−0.810404259*	0.021021955
*Leptotrichia hofstadii*	Post-Tx vs. Pre-Tx (T2DM)	*0.874049907*	0.026491376
*Actinomyces oris*	Post-Tx vs. Pre-Tx (T2DM)	*1.606700209*	0.030890411
*Filifactor alocis*	Post-Tx vs. Pre-Tx (T2DM)	*−0.717132864*	0.033596384
*Bacteroides plebeius*	Post-Tx vs. Pre-Tx (T2DM)	*−0.783873621*	0.04817367
*Muribaculum intestinale*	Post-Tx vs. Pre-Tx (T2DM)	*0.545726021*	0.049357185
Feature	Value	Coef	Pval
*Species significantly differing between Post-Tx groups and Pre-Tx group in the Sal niche*			
*Aureimonas glaciistagni*	Post-Tx vs. Pre-Tx (Periodontitis)	*2.901899735*	0.000125404
*Methylorubrum extorquens*	Post-Tx vs. Pre-Tx (Periodontitis)	*3.063864102*	8.21521E-05
*Sphingomonas faeni*	Post-Tx vs. Pre-Tx (Periodontitis)	*1.942901407*	0.000100676
*Catonella morbi*	Post-Tx vs. Pre-Tx (Periodontitis)	*−1.567313267*	0.000213638
*Phocaeicola abscessus*	Post-Tx vs. Pre-Tx (Periodontitis)	*−2.555974816*	0.000217109
*Faecalibacterium prausnitzii*	Post-Tx vs. Pre-Tx (Periodontitis)	*−3.083813303*	0.000318366
*Peptostreptococcus stomatis*	Post-Tx vs. Pre-Tx (Periodontitis)	*−1.211285008*	0.000443145
*Porphyromonas endodontalis*	Post-Tx vs. Pre-Tx (Periodontitis)	*−1.459019008*	0.000767058
*Filifactor alocis*	Post-Tx vs. Pre-Tx (Periodontitis)	*−2.100952333*	0.001727777
*Parvimonas micra*	Post-Tx vs. Pre-Tx (Periodontitis)	*−1.473846678*	0.001852294
*Eubacterium brachy*	Post-Tx vs. Pre-Tx (Periodontitis)	*−2.054444598*	0.002273795
*Oribacterium sinus*	Post-Tx vs. Pre-Tx (Periodontitis)	*1.675997983*	0.003522328
*Phyllobacterium myrsinacearum*	Post-Tx vs. Pre-Tx (Periodontitis)	*−3.274143501*	0.003343718
*Brevundimonas vesicularis*	Post-Tx vs. Pre-Tx (Periodontitis)	*2.961457553*	0.007829432
*Fusobacterium simiae*	Post-Tx vs. Pre-Tx (Periodontitis)	*−1.910793583*	0.008096217
*Campylobacter gracilis*	Post-Tx vs. Pre-Tx (Periodontitis)	*−1.756687605*	0.015437282
*Prevotella intermedia*	Post-Tx vs. Pre-Tx (Periodontitis)	*−1.390472416*	0.013314138
*Pseudoramibacter alactolyticus*	Post-Tx vs. Pre-Tx (Periodontitis)	*−1.632913082*	0.013945875
*Bacteroides uniformis*	Post-Tx vs. Pre-Tx (Periodontitis)	*−1.095004156*	0.016048528
*Bifidobacterium dentium*	Post-Tx vs. Pre-Tx (Periodontitis)	*1.023501075*	0.024220368
*Fretibacterium fastidiosum*	Post-Tx vs. Pre-Tx (Periodontitis)	*−1.498256465*	0.028479137
*Fusobacterium hwasookii*	Post-Tx vs. Pre-Tx (Periodontitis)	*−1.182421682*	0.030557782
*Fusobacterium massiliense*	Post-Tx vs. Pre-Tx (Periodontitis)	*−0.681938661*	0.028112893
*Lachnospira pectinoschiza*	Post-Tx vs. Pre-Tx (Periodontitis)	*−1.120681448*	0.019036103
*Leptotrichia wadei*	Post-Tx vs. Pre-Tx (Periodontitis)	*1.301153083*	0.028973109
*Oribacterium asaccharolyticum*	Post-Tx vs. Pre-Tx (Periodontitis)	*1.340803987*	0.022130109
*Porphyromonas gingivalis*	Post-Tx vs. Pre-Tx (Periodontitis)	*−1.564828815*	0.017840408
*Stomatobaculum longum*	Post-Tx vs. Pre-Tx (Periodontitis)	*1.333089363*	0.02655596
*Tannerella forsythia*	Post-Tx vs. Pre-Tx (Periodontitis)	*−1.420076638*	0.024480111
*Fusobacterium nucleatum*	Post-Tx vs. Pre-Tx (Periodontitis)	*−1.029868405*	0.032588501
*Peptoanaerobacter stomatis*	Post-Tx vs. Pre-Tx (Periodontitis)	*−1.39158905*	0.033922375
*Eubacterium sulci*	Post-Tx vs. Pre-Tx (Periodontitis)	*−0.931786322*	0.038640886
*Pyramidobacter piscolens*	Post-Tx vs. Pre-Tx (Periodontitis)	*−0.84987103*	0.041497994
*Actinomyces graevenitzii*	Post-Tx vs. Pre-Tx (Periodontitis)	*−0.876810909*	0.042077577
*Faecalibacterium prausnitzii*	Post-Tx vs. Pre-Tx (T2DM)	*−4.613450432*	2.12237E-12
*Phyllobacterium myrsinacearum*	Post-Tx vs. Pre-Tx (T2DM)	*−7.768293199*	1.89328E-12
*Phocaeicola abscessus*	Post-Tx vs. Pre-Tx (T2DM)	*−2.816706269*	1.22166E-11
*Bacteroides ovatus*	Post-Tx vs. Pre-Tx (T2DM)	*−2.653659194*	2.66323E-08
*Lachnoclostridium edouardi*	Post-Tx vs. Pre-Tx (T2DM)	*−2.075840478*	7.4266E-08
*Lachnospira pectinoschiza*	Post-Tx vs. Pre-Tx (T2DM)	*−2.839442842*	3.1917E-07
*Sphingomonas leidyi*	Post-Tx vs. Pre-Tx (T2DM)	*−4.079856107*	3.89091E-07
*Bacteroides uniformis*	Post-Tx vs. Pre-Tx (T2DM)	*−2.149778875*	3.03639E-06
*Prevotella jejuni*	Post-Tx vs. Pre-Tx (T2DM)	*2.542804661*	4.15443E−06
*Odoribacter splanchnicus*	Post-Tx vs. Pre-Tx (T2DM)	*−1.646915917*	6.27029E-05
*Bifidobacterium dentium*	Post-Tx vs. Pre-Tx (T2DM)	*−2.076548025*	0.000104892
*Fusobacterium hwasookii*	Post-Tx vs. Pre-Tx (T2DM)	*1.483135858*	0.000138829
*Bergeyella cardium*	Post-Tx vs. Pre-Tx (T2DM)	*1.517795087*	0.000281928
*Gemmiger formicilis*	Post-Tx vs. Pre-Tx (T2DM)	*−1.680329029*	0.000484402
*Leptotrichia wadei*	Post-Tx vs. Pre-Tx (T2DM)	*−1.673994865*	0.000636321
*Fusobacterium nucleatum*	Post-Tx vs. Pre-Tx (T2DM)	*0.789318977*	0.000678359
*Cardiobacterium hominis*	Post-Tx vs. Pre-Tx (T2DM)	*−1.054978549*	0.001483333
*Porphyromonas catoniae*	Post-Tx vs. Pre-Tx (T2DM)	*1.37192516*	0.001696728
*Nitrospira defluvii*	Post-Tx vs. Pre-Tx (T2DM)	*−1.590792297*	0.001801961
*Eubacterium siraeum*	Post-Tx vs. Pre-Tx (T2DM)	*−1.107567608*	0.002141234
*Parabacteroides merdae*	Post-Tx vs. Pre-Tx (T2DM)	*−0.966554562*	0.00303759
*Bulleidia extructa*	Post-Tx vs. Pre-Tx (T2DM)	*−1.945506731*	0.00399852
*Alistipes obesi*	Post-Tx vs. Pre-Tx (T2DM)	*−0.792923915*	0.009188767
*Prevotella aurantiaca*	Post-Tx vs. Pre-Tx (T2DM)	*−1.06974882*	0.013466729
*Schaalia odontolytica*	Post-Tx vs. Pre-Tx (T2DM)	*−1.069913619*	0.017135864
*Actinomyces oris*	Post-Tx vs. Pre-Tx (T2DM)	*−1.097212463*	0.019026688
*Olsenella uli*	Post-Tx vs. Pre-Tx (T2DM)	*−0.964458492*	0.021239494
*Slackia exigua*	Post-Tx vs. Pre-Tx (T2DM)	*−1.009959556*	0.02526087
*Prevotella intermedia*	Post-Tx vs. Pre-Tx (T2DM)	*−0.879235342*	0.027065757
*Bacteroides caccae*	Post-Tx vs. Pre-Tx (T2DM)	*−0.414801398*	0.03699826
*Mediterraneibacter faecis*	Post-Tx vs. Pre-Tx (T2DM)	*−0.590911233*	0.036765848
*Lachnoanaerobaculum cf.*	Post-Tx vs. Pre-Tx (T2DM)	*−0.463053957*	0.03846214
*Prevotella pallens*	Post-Tx vs. Pre-Tx (T2DM)	*1.000030183*	0.039161626
Feature	Value	Coef	Pval
*Species significantly differing between Post-Tx groups and Pre-Tx group in the TB niche*			
*Phyllobacterium myrsinacearum*	Post-Tx vs. Pre-Tx (Periodontitis)	*5.510759716*	1.63627E-06
*Sphingomonas leidyi*	Post-Tx vs. Pre-Tx (Periodontitis)	*4.380509496*	1.75779E-05
*Lancefieldella parvula*	Post-Tx vs. Pre-Tx (Periodontitis)	*3.060682031*	2.85493E-05
*Brevundimonas vesicularis*	Post-Tx vs. Pre-Tx (Periodontitis)	*3.101917125*	0.000454412
*Lachnoanaerobaculum cf.*	Post-Tx vs. Pre-Tx (Periodontitis)	*−0.902030013*	0.000706272
*Rothia aeria*	Post-Tx vs. Pre-Tx (Periodontitis)	*1.773402806*	0.001242396
*Stomatobaculum longum*	Post-Tx vs. Pre-Tx (Periodontitis)	*2.889959983*	0.001032116
*Solobacterium moorei*	Post-Tx vs. Pre-Tx (Periodontitis)	*0.867807177*	0.002542097
*Prevotella histicola*	Post-Tx vs. Pre-Tx (Periodontitis)	*2.646631691*	0.003248822
*Capnocytophaga sputigena*	Post-Tx vs. Pre-Tx (Periodontitis)	*2.216574018*	0.013699175
*Fretibacterium fastidiosum*	Post-Tx vs. Pre-Tx (Periodontitis)	*1.145868487*	0.020000284
*Porphyromonas pasteri*	Post-Tx vs. Pre-Tx (Periodontitis)	*−1.495641963*	0.03090628
*Prevotella melaninogenica*	Post-Tx vs. Pre-Tx (Periodontitis)	*−0.72356563*	0.041831382
*Schaalia odontolytica*	Post-Tx vs. Pre-Tx (Periodontitis)	*1.818121356*	0.045742189
*Brevundimonas vesicularis*	Post-Tx vs. Pre-Tx (T2DM)	*3.857663335*	5.28276E-08
*Phyllobacterium myrsinacearum*	Post-Tx vs. Pre-Tx (T2DM)	*2.972373044*	0.000192154
*Oribacterium sinus*	Post-Tx vs. Pre-Tx (T2DM)	*−1.039826494*	0.002271253
*Eubacterium sulci*	Post-Tx vs. Pre-Tx (T2DM)	*0.925238554*	0.0070653
*Campylobacter concisus*	Post-Tx vs. Pre-Tx (T2DM)	*−0.85268726*	0.015183978
*Rothia dentocariosa*	Post-Tx vs. Pre-Tx (T2DM)	*1.307062519*	0.014919521
*Prevotella jejuni*	Post-Tx vs. Pre-Tx (T2DM)	*1.50931106*	0.021657029
